# Anticholinergic burden and incident dementia: a Swedish nationwide case-control study

**DOI:** 10.1186/s13195-025-01883-8

**Published:** 2025-10-21

**Authors:** Nanbo Zhu, Maria Eriksdotter, Sophie Liabeuf, Edwin C. K. Tan, Kristina Johnell, Sara Garcia-Ptacek, Hong Xu

**Affiliations:** 1 Department of Neurobiology, Division of Clinical Geriatrics, Care Sciences and Society, Karolinska Institutet, Stockholm, Sweden; 2 Theme Inflammation and Aging, Karolinska University Hospital, Stockholm, Sweden; 3 Department of Clinical Pharmacology, Amiens University Hospital, Amiens, France; 4 MP3CV Laboratory, EA7517, Jules Verne University of Picardie, Amiens, France; 5 Faculty of Medicine and Health, School of Pharmacy, The University of Sydney, Sydney, NSW Australia; 6 Department of Medical Epidemiology and Biostatistics, Karolinska Institutet, Stockholm, Sweden

**Keywords:** Anticholinergic burden, Alzheimer’s disease, Vascular dementia, Lewy body dementia

## Abstract

**Background:**

Cumulative exposure to multiple drugs with anticholinergic properties, known as anticholinergic burden, has been linked to dementia risk. We aimed to thoroughly assess this relationship by examining not only anticholinergic potency, cumulative dose, and drug class, but also dementia subtype and severity.

**Methods:**

This Swedish nationwide case-control study included 199,526 individuals aged ≥ 40 years with incident dementia between July 2008 and December 2017, with 1:1 matched controls based on age, sex, and region of residence. Each case-control group was allocated an identical drug exposure period. Using the Anticholinergic Cognitive Burden (ACB) scale, we separately calculated the total defined daily doses (DDDs) of drugs with weak (ACB score of 1) and strong anticholinergic properties (ACB score of 2–3) prescribed ≥ 1 year before dementia onset. Conditional logistic regression was used to estimate the adjusted odds ratios (AORs) for the association between anticholinergic burden and all-cause dementia, with additional stratified analyses by drug class, dementia subtypes, and severity.

**Results:**

A nonlinear dose-response relationship with all-cause dementia was found for the cumulative use of strong anticholinergics (e.g., 1–89 DDDs: AOR, 1.10 [95% CI, 1.08–1.12]; ≥1095 DDDs: AOR, 1.66 [95% CI, 1.55–1.79]), notably within the class of urinary antispasmodics, antihistamines, and psychotropic drugs. In contrast, a subtle association lacking a dose-response pattern was observed for weak anticholinergics (e.g., 1–89 DDDs: AOR, 1.11 [95% CI, 1.08–1.13]; ≥1095 DDDs: AOR, 1.01 [95% CI, 0.98–1.03]). The associations with strong anticholinergics were more pronounced in men, younger people, those diagnosed with vascular dementia or Lewy body dementia (versus Alzheimer’s disease), and those with milder-stage dementia.

**Conclusions:**

Use of strong anticholinergics, but not weak ones, was associated with a higher risk of dementia, with the strength of association depending on cumulative dose, drug class, and dementia subtype. Caution should be exercised when prescribing drugs with strong anticholinergic properties to middle-aged and older individuals.

**Supplementary Information:**

The online version contains supplementary material available at 10.1186/s13195-025-01883-8.

## Introduction

Dementia prevention through addressing modifiable risk factors across the lifespan remains a key priority for alleviating the growing disease burden [1]. Several factors such as low education, hypertension, and smoking are well established for their detrimental impacts on cognition [1], while the role of potentially inappropriate medications in older adults, particularly those with anticholinergic properties (hereafter referred to as anticholinergic drugs), is not yet fully recognized [2].

The cholinergic system is pivotal in the pathophysiology and treatment of Alzheimer’s disease, as evidenced by the formulation of the “cholinergic hypothesis” and the development of cholinesterase inhibitor therapies [3]. Conversely, anticholinergic drugs inhibit the activity of acetylcholine, a neurotransmitter essential for memory and cognitive function. In clinical use, anticholinergic drugs encompass a broad range of medications with varying levels of anticholinergic activity, commonly prescribed to older people for various conditions. Some drugs (e.g., gastrointestinal and urinary antispasmodics) are intentionally used to block the effects of acetylcholine for therapeutic purposes, whereas many others (e.g., antihistamines, antidepressants, and antipsychotics) can induce anticholinergic adverse drug reactions alongside their primary mechanism of action [4].

Anticholinergic burden, denoting cumulative exposure to multiple drugs with anticholinergic properties, is typically evaluated using expert consensus-based scales [5]. Accumulating literature has linked greater anticholinergic burden to increased risks of cognitive impairment and dementia [6–12]. However, most previous research has been rated as having a high risk of bias [6–8], often suffering from potential reverse causation, inadequate control for confounding, and inclusion of prevalent users, contributing to the overall low certainty of evidence [8]. In addition, few studies have delved into comparing associations by the drugs’ anticholinergic potency [11], examining dose-response relationships [10–12], or unraveling drug class-specific associations [11, 12]. Further investigations are also needed to understand how these associations vary across different subtypes or severity of dementia. Such finer-grained analyses could provide more insight into the putative links and offer valuable guidance for clinical practice, but require a large sample size, sufficient drug exposure duration, and detailed diagnostic information.

Using Swedish population-based register data, we conducted a nationwide case-control study to comprehensively examine the associations between anticholinergic burden and risk of dementia, considering anticholinergic potency, cumulative dose, and drug class, as well as dementia subtype and severity.

## Methods

### Data source

This study was based on the linkage of multiple Swedish national registers through unique personal identification numbers. The Swedish Registry for Cognitive/Dementia Disorders (SveDem) is a national quality registry initiated in 2007 that enrolls patients diagnosed with incident dementia from either primary care or specialist memory clinics and collects diagnostic information, including date of diagnosis, type of dementia, and severity of dementia assessed by the Mini-Mental State Examination (MMSE) score [13]. The Total Population Register provided demographic information including age, sex, and place of residence [14]; the National Patient Register provided clinical diagnoses coded according to the 10th revision of the International Classification of Diseases from hospital admissions since 1997 and from outpatient specialist consultations since 2001 [15]; the Cause of Death Register provided information on the dates and underlying causes of all deaths in Sweden [16]; the Prescribed Drug Register provided complete information on all prescribed drugs dispensed at Swedish pharmacies since July 2005, including details such as date of prescribing and dispensing, drug’s identity categorized by the Anatomical Therapeutic Chemical classification system, amount, and dosage [17]; the Longitudinal Integrated Database for Health Insurance and Labor Market Studies provided socioeconomic characteristics including educational attainment, disposable income, marital status, and region of birth [18].

This study was approved by the regional ethics committee in Stockholm (Dnr: 2017/501 − 31; 2017/1448-32; 2021–05289) and adhered to the Declaration of Helsinki. Participants were informed about their registration in SveDem and could decline participation or withdraw registration. Informed consent is not required for pseudo-anonymized register-based research according to Swedish law. We have followed the Reporting of Studies Conducted Using Observational Routinely Collected Health Data for Pharmacoepidemiology (RECORD-PE) guideline [19].

### Study design

The study design is graphically depicted in Fig. S1. We conducted a nationwide case-control study in Sweden that included all individuals aged ≥ 40 years with incident dementia between July 1, 2008, and December 31, 2017. The index date was defined as the date of incident dementia for case patients and was assigned to their matched controls. Given the establishment of the Swedish Prescribed Drug Register in July 2005 [17], we set the baseline as July 2006, allowing for a one-year washout period. Accordingly, each individual was ensured a minimum follow-up of two years, comprising an at least one-year exposure period to assess the anticholinergic burden and a one-year lag period to reduce protopathic bias [10, 12]. A prior study based on SveDem showed a marked increase in the use of psychotropic medications (e.g., antidepressants, anxiolytics, and antipsychotics) starting approximately 1 to 2 years before receipt of dementia diagnosis, compared to individuals without dementia [20].

### Selection of cases and controls

A flowchart for the sample selection is shown in Fig. S2. Cases with incident dementia were identified through several data sources, including a new diagnosis of dementia from SveDem, the National Patient Register, and the Cause of Death Register, as well as the initiation of anti-dementia drugs recorded in the Prescribed Drug Register (Table S1). Control individuals, who remained free of dementia throughout the study period, were drawn from the Total Population Register. For each patient with dementia, up to 4 controls were randomly selected based on year of birth (± 3 years), sex, and region of residence. To mimic a new-user design, cases and controls were excluded if they had used drugs with strong anticholinergic effects (as defined below) during the washout period. Thereafter, we randomly selected one control for each case to maintain balance in the matching variables.

### Exposures

Drugs considered as having anticholinergic properties were determined using the Anticholinergic Cognitive Burden (ACB) scale [21], which is the most commonly used tool to measure anticholinergic burden and has been ranked highest in a systematic quality assessment [5]. The scale’s scoring system classifies drugs according to their anticholinergic potency (Table S2): 0 points indicate no known anticholinergic effects, 1 point indicates possibly anticholinergic effects (demonstrating serum anticholinergic activity or in vitro affinity to muscarinic receptors but lacking clinically relevant negative cognitive effects), and 2 or 3 points indicate definitely anticholinergic effects with established clinical relevance.

Each case-control group, by design, was allocated an identical drug exposure period, starting at baseline and ending one year before the index date. Prescriptions dispensed within the year preceding the index date were not included to mitigate protopathic bias. The main exposures considered both the potency and cumulative doses of anticholinergics. We calculated cumulative defined daily doses (DDDs) separately for drugs classified as weak anticholinergics (ACB score = 1) and strong anticholinergics (ACB score = 2 or 3) by totaling DDDs prescribed within each potency category over the exposure assessment period, which were categorized into 0, 1–89, 90–364, 365–1094, or ≥ 1095 DDDs for clinical interpretability [10, 12].

In further analysis, we categorized drug classes according to the Anatomical Therapeutic Chemical code (Table S3). The highest exposure category in the stratified analyses by drug class, dementia subtype, or MMSE score was defined as ≥ 365 DDDs to account for the limited number of individuals.

### Covariates

Potential confounders included risk factors for dementia and indications for anticholinergic drugs. In the primary analysis, covariates were ascertained at baseline, including sociodemographic factors, healthcare utilization in the previous year, physical and neuropsychiatric comorbidities, and use of medications not listed on the ACB scale within the previous six months (see covariates listed in Table 1). Definitions of comorbidities and medications are shown in Table S4.

**Table 1:** Baseline characteristics of dementia cases and matched controls

Characteristics	Dementia cases *N* = 199,526	Controls *N* = 199,526
Age, median (IQR), y	77 (70–82)	77 (70–82)
Female	116,321 (58.3)	116,321 (58.3)
Disposable personal income,^*^ median (IQR), ×100 SEK	1178 (964–1496)	1196 (969–1566)
Educational attainment		
Compulsory education	103,098 (51.7)	99,575 (49.9)
Upper secondary education	63,612 (31.9)	64,231 (32.2)
University/college	27,318 (13.7)	30,843 (15.5)
Missing	5498 (2.8)	4877 (2.4)
Marital status ^*^		
Single	16,764 (8.5)	15,249 (7.7)
Married	98,498 (49.7)	103,996 (52.4)
Divorced	26,802 (13.5)	23,633 (11.9)
Widowed	56,074 (28.3)	55,704 (28.1)
Cohabitation status ^*^		
Cohabitation	98,685 (49.8)	104,104 (52.4)
Living alone	99,451 (50.2)	94,478 (47.6)
Region of birth ^*^		
Sweden	176,731 (89.2)	178,810 (90.0)
Other countries	21,407 (10.8)	19,772 (10.0)
Any outpatient visit in the past year	93,462 (46.8)	90,332 (45.3)
Any hospitalization in the past year	33,156 (16.6)	29,348 (14.7)
Physical comorbidities		
Hypertension	106,222 (53.2)	107,256 (53.8)
Diabetes mellitus	22,616 (11.3)	18,383 (9.2)
Osteoarthritis	20,065 (10.1)	20,176 (10.1)
Stroke	18,358 (9.2)	13,665 (6.8)
Atrial fibrillation	16,057 (8.0)	15,368 (7.7)
Cancer	15,203 (7.6)	16,458 (8.2)
Myocardial infarction	11,854 (5.9)	11,979 (6.0)
Congestive heart failure	9528 (4.8)	9775 (4.9)
Dyslipidemia	8776 (4.4)	8097 (4.1)
Hearing loss	7558 (3.8)	7203 (3.6)
Lung disease	7177 (3.6)	7362 (3.7)
Rheumatic disease	6641 (3.3)	6656 (3.3)
Chronic pain	6386 (3.2)	5826 (2.9)
Back pain	6362 (3.2)	5755 (2.9)
Gastroesophageal reflux disease	4896 (2.5)	4800 (2.4)
Peripheral vascular disease	4537 (2.3)	4564 (2.3)
Vestibular disorder	4499 (2.3)	4027 (2.0)
Peptic ulcer disease	3896 (2.0)	3430 (1.7)
Urinary incontinence	3586 (1.8)	3228 (1.6)
Liver disease	1256 (0.6)	978 (0.5)
Kidney disease	1211 (0.6)	1338 (0.7)
Inflammatory bowel disease	1183 (0.6)	1196 (0.6)
Irritable bowel syndrome	744 (0.4)	643 (0.3)
Hemiplegia/paraplegia	506 (0.3)	387 (0.2)
Neuropsychiatric comorbidities		
Depression	3950 (2.0)	2263 (1.1)
Migraine/headache	3487 (1.7)	2778 (1.4)
Substance use disorder	2932 (1.5)	1182 (0.6)
Parkinson’s disease	2163 (1.1)	629 (0.3)
Sleep disorders	1799 (0.9)	1726 (0.9)
Anxiety disorders	1626 (0.8)	1109 (0.6)
Epilepsy	1384 (0.7)	714 (0.4)
Psychotic disorders	1316 (0.7)	577 (0.3)
Stress-related disorders	656 (0.3)	438 (0.2)
Co-medications ^†^		
Antiplatelet drugs	60,121 (30.1)	55,486 (27.8)
Beta-blockers	59,889 (30.0)	61,664 (30.9)
Diuretics	50,980 (25.6)	53,874 (27.0)
Renin-angiotensin system inhibitors	43,963 (22.0)	46,173 (23.1)
Anxiolytics, hypnotics, and sedatives	40,945 (20.5)	37,212 (18.7)
Analgesics	40,724 (20.4)	37,536 (18.8)
Statins	39,725 (19.9)	37,965 (19.0)
Calcium channel blockers	29,795 (14.9)	30,916 (15.5)
Nonsteroidal anti-inflammatory drugs	27,344 (13.7)	29,018 (14.5)
Proton-pump inhibitors	21,992 (11.0)	23,199 (11.6)
Antidepressants	20,856 (10.5)	12,477 (6.3)
Antihistamines	5127 (2.6)	5282 (2.6)
Anti-Parkinson drugs	4299 (2.2)	2261 (1.1)
Antipsychotics	4102 (2.1)	1581 (0.8)
Antispasmodics	3256 (1.6)	3251 (1.6)
Antiepileptics	2608 (1.3)	1831 (0.9)

Values represent *n* (%) unless otherwise indicated

*Abbreviations*: *IQR *interquartile range, *SEK *Swedish krona

^*^ 0.7% of dementia cases and 0.5% of matched controls had missing data

^†^ Drug agents listed on the Anticholinergic Cognitive Burden scale were not included in the count

### Statistical analysis

We used conditional logistic regression to estimate odds ratios (ORs) and 95% confidence intervals (CIs) for the risk of all-cause dementia associated with cumulative DDDs (in five categories) prescribed for weak anticholinergic drugs and strong anticholinergic drugs. Stepwise, model 1 served as a crude model but inherently accounted for the matching variables; model 2 was adjusted for sociodemographics, prior healthcare utilization, and comorbidities; model 3 was additionally adjusted for other medication use. Cumulative use of anticholinergic drugs was also modeled as continuous measures using restricted cubic splines to delineate potential nonlinear relationships. Furthermore, we examined associations specific to certain classes of anticholinergics within each potency level.

We performed pre-specified subgroup analysis according to sex (female or male) and age at diagnosis (< 65, 65–74, 75–84, or ≥ 85 years). In addition, we evaluated the associations of anticholinergic burden with specific types of dementia (Alzheimer’s disease/mixed dementia, vascular dementia, Lewy body dementia, or frontotemporal dementia). Among cases identified from SveDem, we further conducted stratified analyses by MMSE score at diagnosis (0–9: severe, 10–19: moderate, 20–24: mild, or 25–30: very mild stage).

To test the robustness of our results, we performed several sensitivity analyses. Given the importance of addressing protopathic bias, we extended the 1-year lag period to 3 and 5 years to further reduce potential bias for the above analyses. Other sensitivity analyses focused on the overall anticholinergic burden and all-cause dementia. First, we repeated the main analysis using the entire control sample. Second, we employed the recently developed Swedish anticholinergic burden scale to identify drugs with anticholinergic properties [22], which is adapted to drugs marketed in Sweden but has not yet been validated. Third, we implemented alternative adjustments for covariates, including (1) additionally adjusting for multimorbidity evaluated by the Charlson comorbidity index and polypharmacy measured by the count of medications, (2) additionally adjusting for cumulative use of medications considered as baseline covariates during the exposure period, and (3) adjusting for all covariates ascertained at the index date rather than at baseline. Finally, we included users of strong anticholinergic drugs during the washout period because it is unclear how the inclusion or exclusion of prevalent users influences the estimation of long-term effects [23]. All analyses were performed using R version 4.3.1 (R Foundation for Statistical Computing). A two-tailed *P* < 0.001 was chosen as the threshold for statistical significance to accommodate multiple subgroup analyses.

## Results

### Study population

The analysis consisted of 199,526 patients with incident dementia, matched with an equivalent number of controls. The median (interquartile range) age was 77 (70–82) years at baseline and 84 (78–88) at diagnosis; 116,321 (58.3%) were women. The prevalence of comorbidities and prescribed medications was comparable or slightly higher among cases than controls (Table 1).

During a median drug exposure period of 5.9 years (interquartile range 3.5–8.2), a total of 634,427 prescriptions for strong anticholinergic drugs (ACB score = 2 or 3) and 10,573,038 prescriptions for weak anticholinergic drugs (ACB score = 1) were recorded. Figs. S3–S6 present the number of prescriptions and DDDs by drug class, or by individual drug. The most frequently prescribed types of strong anticholinergic drugs were anxiolytics (24.9%), urinary antispasmodics (24.7%), and antidepressants (16.4%), while prescriptions of weak anticholinergic drugs were predominantly comprised of cardiovascular agents (76.2%), followed by antithrombotic agents (7.7%), and analgesics (5.0%) (Fig. S3).

### Anticholinergic burden and incident dementia

**Fig. 1:**
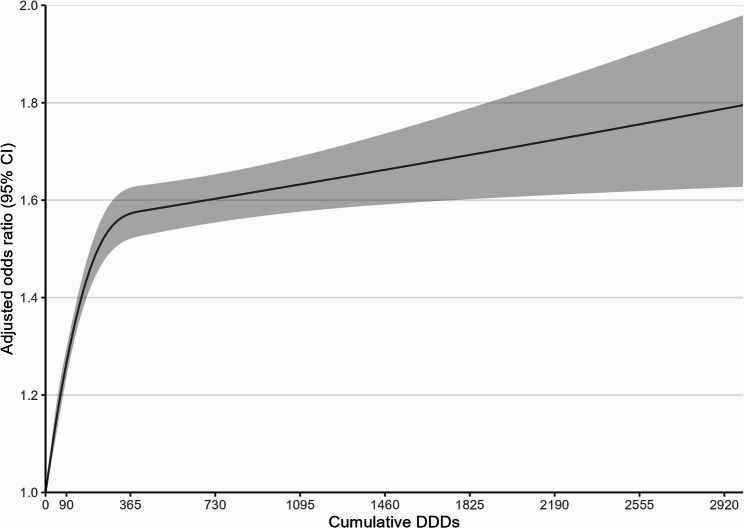
Association between cumulative use of strong anticholinergic drugs and all-cause dementia using restricted cubic splines. Abbreviations: CI, confidence interval; DDD, defined daily dose. The cubic spline was constructed with knots placed at the 5th, 35th, 65th, and 95th percentiles of cumulative doses. The solid line represents the adjusted odds ratios, and the shaded area represents the corresponding 95% CIs

21.3% of case patients and 17.4% of controls were prescribed at least one strong anticholinergic drug, while 70.3% of case patients and 68.2% of controls were prescribed at least one weak anticholinergic drug (Table 2). The crude associations were attenuated following stepwise adjustments. Applying longer lag periods resulted in smaller sample sizes but similar associations. In the final model with 1-year lag time, the adjusted OR (95% CI) associated with cumulative exposure to strong anticholinergic drugs increased from 1.10 (1.08, 1.12) for 1–89 DDDs to 1.39 (1.34, 1.43) for 90–364 DDDs, 1.57 (1.50, 1.65) for 365–1094 DDDs, and 1.66 (1.55, 1.79) for ≥ 1095 DDDs. As illustrated in Fig. 1, an evident nonlinear dose-response relationship was observed, with the OR increasing rapidly until 365 DDDs and then exhibiting a slower increase. In contrast, cumulative exposure to weak anticholinergic drugs, compared with nonuse, was only associated with a slightly higher risk of dementia, except for the category of ≥ 1095 DDDs (OR, 1.01; 95% CI, 0.98–1.03). Analysis with restricted cubic spline demonstrated a peak OR of 1.1 at around 365–730 DDDs, after which it diminished to null (Fig. S7).

**Table 2:** Association between cumulative use of anticholinergic drugs and all-cause dementia

Exposure, DDDs	No. of cases (%)	No. of controls (%)	Odds ratio (95% CI)^*^		
			Model 1	Model 2	Model 3
1-year lag time					
Strong anticholinergics					
0	157,103 (78.7)	164,771 (82.6)	1.00	1.00	1.00
1–89	24,856 (12.5)	23,161 (11.6)	1.12 (1.10, 1.14)^‡^	1.10 (1.08, 1.13)^‡^	1.10 (1.08, 1.12)^‡^
90–364	10,673 (5.3)	7538 (3.8)	1.48 (1.43, 1.52)^‡^	1.42 (1.38, 1.47)^‡^	1.39 (1.34, 1.43)^‡^
365–1094	4763 (2.4)	2880 (1.4)	1.74 (1.66, 1.82)^‡^	1.62 (1.55, 1.70)^‡^	1.57 (1.50, 1.65)^‡^
≥ 1095	2131 (1.1)	1176 (0.6)	1.91 (1.78, 2.05)^‡^	1.73 (1.61, 1.87)^‡^	1.66 (1.55, 1.79)^‡^
Weak anticholinergics					
0	59,225 (29.7)	63,384 (31.8)	1.00	1.00	1.00
1–89	21,236 (10.6)	19,708 (9.9)	1.13 (1.10, 1.15)^‡^	1.12 (1.09, 1.15)^‡^	1.11 (1.08, 1.13)^‡^
90–364	23,546 (11.8)	21,563 (10.8)	1.13 (1.11, 1.16)^‡^	1.13 (1.10, 1.15)^‡^	1.11 (1.09, 1.14)^‡^
365–1094	34,309 (17.2)	32,962 (16.5)	1.09 (1.06, 1.11)^‡^	1.07 (1.05, 1.10)^‡^	1.06 (1.04, 1.08)^‡^
≥ 1095	61,210 (30.7)	61,909 (31.0)	1.03 (1.01, 1.04)^†^	1.00 (0.98, 1.02)	1.01 (0.98, 1.03)
3-year lag time					
Strong anticholinergics					
0	132,183 (82.6)	136,888 (85.5)	1.00	1.00	1.00
1–89	17,239 (10.8)	16,059 (10.0)	1.11 (1.08, 1.13)^‡^	1.09 (1.06, 1.11)^‡^	1.08 (1.05, 1.11)^‡^
90–364	6663 (4.2)	4722 (3.0)	1.45 (1.40, 1.51)^‡^	1.39 (1.33, 1.44)^‡^	1.35 (1.30, 1.41)^‡^
365–1094	2835 (1.8)	1775 (1.1)	1.65 (1.55, 1.75)^‡^	1.53 (1.44, 1.62)^‡^	1.47 (1.38, 1.57)^‡^
≥ 1095	1115 (0.7)	591 (0.4)	1.94 (1.76, 2.15)^‡^	1.74 (1.57, 1.93)^‡^	1.66 (1.50, 1.84)^‡^
Weak anticholinergics					
0	57,026 (35.6)	60,219 (37.6)	1.00	1.00	1.00
1–89	16,093 (10.1)	15,505 (9.7)	1.08 (1.05, 1.10)^‡^	1.07 (1.04, 1.09)^‡^	1.06 (1.03, 1.08)^‡^
90–364	18,949 (11.8)	17,850 (11.2)	1.10 (1.07, 1.12)^‡^	1.08 (1.05, 1.10)^‡^	1.06 (1.04, 1.09)^‡^
365–1094	28,282 (17.7)	27,484 (17.2)	1.07 (1.05, 1.09)^‡^	1.04 (1.02, 1.07)^‡^	1.03 (1.01, 1.06)^†^
≥ 1095	39,685 (24.8)	38,977 (24.4)	1.06 (1.04, 1.08)^‡^	1.00 (0.98, 1.02)	1.00 (0.98, 1.03)
5-year lag time					
Strong anticholinergics					
0	101,692 (86.2)	104,378 (88.4)	1.00	1.00	1.00
1–89	10,643 (9.0)	9824 (8.3)	1.10 (1.07, 1.13)^‡^	1.08 (1.04, 1.11)^‡^	1.06 (1.03, 1.10)^‡^
90–364	3735 (3.2)	2676 (2.3)	1.42 (1.35, 1.49)^‡^	1.34 (1.27, 1.41)^‡^	1.30 (1.24, 1.37)^‡^
365–1094	1499 (1.3)	893 (0.8)	1.70 (1.56, 1.85)^‡^	1.55 (1.43, 1.69)^‡^	1.49 (1.37, 1.63)^‡^
≥ 1095	445 (0.4)	243 (0.2)	1.86 (1.59, 2.18)^‡^	1.67 (1.42, 1.95)^‡^	1.58 (1.35, 1.86)^‡^
Weak anticholinergics					
0	48,932 (41.5)	51,692 (43.8)	1.00	1.00	1.00
1–89	11,229 (9.5)	10,702 (9.1)	1.09 (1.06, 1.13)^‡^	1.08 (1.05, 1.11)^‡^	1.07 (1.04, 1.10)^‡^
90–364	14,399 (12.2)	13,767 (11.7)	1.09 (1.06, 1.12)^‡^	1.06 (1.03, 1.09)^‡^	1.05 (1.02, 1.08)^†^
365–1094	21,219 (18.0)	20,599 (17.5)	1.08 (1.05, 1.10)^‡^	1.04 (1.01, 1.06)^†^	1.03 (1.00, 1.05)
≥ 1095	22,235 (18.8)	21,254 (18.0)	1.09 (1.07, 1.12)^‡^	1.01 (0.98, 1.04)	1.01 (0.98, 1.04)

*Abbreviations:* *CI *confidence interval, *DDD *defined daily dose

^*^ Model 1 was unadjusted

Model 2 was adjusted for sociodemographic factors, healthcare utilization in the previous year, and history of physical and neuropsychiatric comorbidities

Model 3 was additionally adjusted for the use of medications other than anticholinergics

^†^ *p* < 0.01

^‡^ *p* < 0.001

The association between cumulative use of strong anticholinergic drugs and dementia risk was stronger among men than women (Table S5). There were stronger associations for both strong and weak anticholinergics in patients diagnosed at an earlier age (Table S6). For example, the adjusted OR (95% CI) for ≥ 1095 DDDs of strong anticholinergic drugs was 3.47 (2.15, 5.58) in cases diagnosed before age 65 years, compared with 1.19 (1.06, 1.35) in cases diagnosed after 85 years.

For specific classes of strong anticholinergics, there were significant dose-response associations with the cumulative use of urinary antispasmodics, antihistamines, and all psychotropic medications, but not with gastrointestinal and musculoskeletal system drugs (Table 3). Results were consistent across the different lag periods examined. For specific classes of weak anticholinergics, positive associations were observed with analgesics, antipsychotics, and antidepressants, while negative associations were noted with gastrointestinal drugs, cardiovascular drugs, and antihistamines when using a 1-year lag time (Table S7).

**Table 3:** Association between cumulative use of specific classes of strong anticholinergic drugs and all-cause dementia

Exposure, DDDs	No. of cases (%)	No. of controls (%)	Adjusted odds ratio (95% CI)^*^		
			1-year lag	3-year lag	5-year lag
Gastrointestinal drugs					
0	198,622 (99.5)	198,574 (99.5)	1.00	1.00	1.00
1–89	789 (0.4)	859 (0.4)	0.86 (0.78, 0.95)^†^	0.92 (0.82, 1.04)	0.93 (0.80, 1.08)
90–364	89 (< 0.1)	80 (< 0.1)	0.94 (0.69, 1.28)	0.76 (0.51, 1.13)	0.90 (0.54, 1.50)
≥ 365	26 (< 0.1)	13 (< 0.1)	1.67 (0.83, 3.36)	–	–
Urinary antispasmodics					
0	187,796 (94.1)	190,479 (95.5)	1.00	1.00	1.00
1–89	4070 (2.0)	3744 (1.9)	1.08 (1.03, 1.13)^†^	1.13 (1.07, 1.19)^‡^	1.12 (1.04, 1.20)^†^
90–364	4066 (2.0)	3024 (1.5)	1.32 (1.25, 1.38)^‡^	1.32 (1.24, 1.40)^‡^	1.24 (1.15, 1.34)^‡^
≥ 365	3594 (1.8)	2279 (1.1)	1.51 (1.43, 1.59)^‡^	1.47 (1.38, 1.58)^‡^	1.52 (1.38, 1.67)^‡^
Muscle relaxants					
0	198,378 (99.4)	198,317 (99.4)	1.00	1.00	1.00
1–89	979 (0.5)	1041 (0.5)	0.90 (0.82, 0.99)	0.97 (0.87, 1.08)	0.98 (0.84, 1.13)
90–364	137 (0.1)	134 (0.1)	0.94 (0.74, 1.20)	1.02 (0.76, 1.39)	0.98 (0.67, 1.45)
≥ 365	32 (< 0.1)	34 (< 0.1)	0.75 (0.45, 1.24)	0.66 (0.33, 1.32)	0.57 (0.22, 1.49)
Antihistamines					
0	188,357 (94.4)	189,085 (94.8)	1.00	1.00	1.00
1–89	8300 (4.2)	8433 (4.2)	0.95 (0.92, 0.98)^†^	0.95 (0.92, 0.99)	0.97 (0.93, 1.02)
90–364	2164 (1.1)	1591 (0.8)	1.18 (1.10, 1.26)^‡^	1.27 (1.16, 1.39)^‡^	1.31 (1.16, 1.48)^‡^
≥ 365	705 (0.4)	417 (0.2)	1.21 (1.07, 1.38)^†^	1.16 (0.98, 1.38)	1.01 (0.78, 1.31)
Antiepileptics					
0	197,389 (98.9)	198,447 (99.5)	1.00	1.00	1.00
1–89	1221 (0.6)	713 (0.4)	1.53 (1.39, 1.69)^‡^	1.45 (1.29, 1.63)^‡^	1.43 (1.23, 1.67)^‡^
90–364	547 (0.3)	207 (0.1)	2.26 (1.91, 2.66)^‡^	1.88 (1.53, 2.30)^‡^	2.06 (1.55, 2.73)^‡^
≥ 365	369 (0.2)	159 (0.1)	1.97 (1.62, 2.38)^‡^	1.99 (1.54, 2.59)^‡^	2.17 (1.48, 3.16)^‡^
Anti-Parkinson drugs					
0	199,178 (99.8)	199,463 (100.0)	1.00	1.00	1.00
1–89	99 (< 0.1)	30 (< 0.1)	2.06 (1.33, 3.18)^†^	2.88 (1.70, 4.88)^‡^	2.64 (1.25, 5.58)
90–364	131 (0.1)	19 (< 0.1)	4.01 (2.42, 6.64)^‡^	3.11 (1.53, 6.30)^†^	3.18 (1.15, 8.74)
≥ 365	118 (0.1)	14 (< 0.1)	3.90 (2.20, 6.92)^‡^	4.28 (1.79, 10.19)^†^	4.81 (1.42, 16.27)
Antipsychotics					
0	196,421 (98.4)	198,690 (99.6)	1.00	1.00	1.00
1–89	2011 (1.0)	572 (0.3)	2.59 (2.36, 2.86)^‡^	1.90 (1.67, 2.16)^‡^	1.59 (1.33, 1.90)^‡^
90–364	676 (0.3)	156 (0.1)	3.01 (2.52, 3.61)^‡^	2.42 (1.88, 3.11)^‡^	1.81 (1.32, 2.49)^‡^
≥ 365	418 (0.2)	108 (0.1)	2.37 (1.90, 2.95)^‡^	2.01 (1.50, 2.71)^‡^	1.81 (1.16, 2.82)^†^
Anxiolytics					
0	183,813 (92.1)	187,419 (93.9)	1.00	1.00	1.00
1–89	12,414 (6.2)	10,102 (5.1)	1.18 (1.15, 1.22)^‡^	1.15 (1.11, 1.19)^‡^	1.13 (1.08, 1.18)^‡^
90–364	2554 (1.3)	1579 (0.8)	1.43 (1.33, 1.52)^‡^	1.27 (1.17, 1.39)^‡^	1.25 (1.11, 1.41)^‡^
≥ 365	745 (0.4)	426 (0.2)	1.39 (1.23, 1.58)^‡^	1.33 (1.12, 1.57)^†^	1.15 (0.88, 1.51)
Antidepressants					
0	192,714 (96.6)	193,447 (97.0)	1.00	1.00	1.00
1–89	4540 (2.3)	4499 (2.3)	0.95 (0.91, 0.99)	0.97 (0.92, 1.02)	1.00 (0.94, 1.07)
90–364	1528 (0.8)	1101 (0.6)	1.17 (1.08, 1.27)^‡^	1.29 (1.17, 1.44)^‡^	1.20 (1.05, 1.38)^†^
≥ 365	744 (0.4)	479 (0.2)	1.25 (1.11, 1.41)^‡^	1.21 (1.04, 1.42)	1.42 (1.14, 1.77)^†^

Numbers of cases and controls are presented for the analysis using a 1-year lag time in the exposure assessment

*Abbreviations:* *CI *confidence interval, *DDD *defined daily dose

^*^ Models were adjusted for sociodemographic factors, healthcare utilization in the previous year, history of physical and neuropsychiatric comorbidities, and use of medications other than anticholinergics

^†^ *p* < 0.01

^‡^ *p* < 0.001

Regarding type-specific dementia, stronger associations with strong anticholinergic drugs were observed for vascular dementia and Lewy body dementia than for Alzheimer’s disease/mixed dementia, although the use of longer lag periods led to greater attenuation of the ORs for the former two subtypes (Table 4). Cumulative use of weak anticholinergic drugs was associated with a higher risk of vascular dementia but a lower risk of Alzheimer’s disease/mixed dementia (Table S8).

**Table 4:** Association between cumulative use of strong anticholinergic drugs and type-specific dementia

Exposure, DDDs	No. of cases (%)	No. of controls (%)	Adjusted odds ratio (95% CI)^*^		
			1-year lag	3-year lag	5-year lag
Alzheimer’s disease/Mixed dementia					
0	36,361 (80.0)	37,414 (82.3)	1.00	1.00	1.00
1–89	5702 (12.5)	5453 (12.0)	1.09 (1.05, 1.14)^‡^	1.09 (1.03, 1.14)^‡^	1.06 (1.00, 1.13)
90–364	2160 (4.7)	1694 (3.7)	1.33 (1.25, 1.43)^‡^	1.35 (1.24, 1.46)^‡^	1.32 (1.18, 1.47)^‡^
≥ 365	1256 (2.8)	918 (2.0)	1.43 (1.31, 1.57)^‡^	1.39 (1.24, 1.57)^‡^	1.37 (1.16, 1.62)^‡^
Vascular dementia					
0	19,364 (77.0)	20,698 (82.3)	1.00	1.00	1.00
1–89	3302 (13.1)	2949 (11.7)	1.13 (1.07, 1.20)^‡^	1.11 (1.04, 1.19)^†^	1.14 (1.05, 1.24)^†^
90–364	1452 (5.8)	969 (3.9)	1.40 (1.28, 1.53)^‡^	1.45 (1.29, 1.61)^‡^	1.40 (1.21, 1.63)^‡^
≥ 365	1024 (4.1)	526 (2.1)	1.75 (1.56, 1.96)^‡^	1.54 (1.33, 1.78)^‡^	1.56 (1.26, 1.92)^‡^
Lewy body dementia					
0	2972 (69.2)	3576 (83.3)	1.00	1.00	1.00
1–89	662 (15.4)	466 (10.9)	1.59 (1.36, 1.86)^‡^	1.44 (1.20, 1.73)^‡^	1.44 (1.15, 1.82)^†^
90–364	383 (8.9)	152 (3.5)	2.55 (2.02, 3.24)^‡^	2.06 (1.53, 2.78)^‡^	1.93 (1.32, 2.80)^‡^
≥ 365	276 (6.4)	99 (2.3)	2.30 (1.73, 3.04)^‡^	1.76 (1.24, 2.51)^†^	1.16 (0.72, 1.88)
Frontotemporal dementia					
0	1323 (77.9)	1437 (84.6)	1.00	1.00	1.00
1–89	232 (13.7)	169 (9.9)	1.44 (1.14, 1.80)^†^	1.07 (0.81, 1.41)	1.07 (0.75, 1.52)
90–364	91 (5.4)	56 (3.3)	1.73 (1.19, 2.53)^†^	1.19 (0.78, 1.81)	0.70 (0.36, 1.33)
≥ 365	53 (3.1)	37 (2.2)	1.32 (0.82, 2.12)	0.86 (0.46, 1.60)	1.75 (0.61, 5.03)

Analyses were performed among individuals with a specific type of dementia and their matched controls. Numbers of cases and controls are presented for the analysis using a 1-year lag time in the exposure assessment

*Abbreviations:* *CI *confidence interval, *DDD *defined daily dose

^*^ Models were adjusted for sociodemographic factors, healthcare utilization in the previous year, history of physical and neuropsychiatric comorbidities, and use of medications other than anticholinergics

^†^ *p* < 0.01

^‡^ *p* < 0.001

When stratifying by MMSE score, there were stronger positive associations with strong anticholinergic drugs in patients with a higher MMSE score, which was consistent across all three lag periods (Table 5), as well as tentative negative associations with weak anticholinergic drugs in patients with a lower MMSE score (Table S9).

**Table 5:** Association between cumulative use of strong anticholinergic drugs and all-cause dementia, by MMSE score at diagnosis

Exposure, DDDs	No. of cases (%)	No. of controls (%)	Adjusted odds ratio (95% CI)^*^		
			1-year lag	3-year lag	5-year lag
MMSE score 25–30					
0	11,327 (77.5)	12,070 (82.5)	1.00	1.00	1.00
1–89	1971 (13.5)	1733 (11.9)	1.18 (1.09, 1.27)^‡^	1.09 (1.00, 1.20)	1.12 (1.00, 1.25)
90–364	774 (5.3)	543 (3.7)	1.43 (1.27, 1.61)^‡^	1.48 (1.28, 1.72)^‡^	1.36 (1.12, 1.66)^†^
≥ 365	551 (3.8)	277 (1.9)	2.03 (1.74, 2.37)^‡^	1.74 (1.42, 2.14)^‡^	2.17 (1.61, 2.93)^‡^
MMSE score 20–24					
0	18,334 (77.7)	19,256 (81.7)	1.00	1.00	1.00
1–89	3181 (13.5)	2840 (12.0)	1.16 (1.09, 1.22)^‡^	1.13 (1.05, 1.20)^‡^	1.08 (0.99, 1.17)
90–364	1276 (5.4)	945 (4.0)	1.39 (1.27, 1.52)^‡^	1.39 (1.25, 1.55)^‡^	1.36 (1.18, 1.56)^‡^
≥ 365	792 (3.4)	542 (2.3)	1.50 (1.33, 1.68)^‡^	1.49 (1.29, 1.73)^‡^	1.53 (1.24, 1.89)^‡^
MMSE score 10–19					
0	15,051 (79.2)	15,360 (80.9)	1.00	1.00	1.00
1–89	2416 (12.7)	2413 (12.7)	1.04 (0.98, 1.11)	1.06 (0.98, 1.14)	1.04 (0.95, 1.14)
90–364	937 (4.9)	784 (4.1)	1.20 (1.08, 1.33)^‡^	1.24 (1.09, 1.41)^‡^	1.12 (0.96, 1.32)
≥ 365	594 (3.1)	441 (2.3)	1.33 (1.16, 1.51)^‡^	1.21 (1.02, 1.42)	1.32 (1.03, 1.69)
MMSE score 0–9					
0	1310 (82.1)	1309 (82.0)	1.00	1.00	1.00
1–89	174 (10.9)	204 (12.8)	0.83 (0.65, 1.06)	0.74 (0.55, 0.99)	0.75 (0.52, 1.09)
90–364	70 (4.4)	56 (3.5)	1.31 (0.85, 1.99)	1.46 (0.87, 2.47)	1.45 (0.66, 3.20)
≥ 365	42 (2.6)	27 (1.7)	1.37 (0.77, 2.43)	1.14 (0.52, 2.49)	0.64 (0.22, 1.85)

Analyses were performed among dementia patients with an available MMSE score and their matched controls. Numbers of cases and controls are presented for the analysis using a 1-year lag time in the exposure assessment

*Abbreviations:* *CI *confidence interval, *DDD *defined daily dose, *MMSE *Mini-Mental State Examination

^*^ Models were adjusted for sociodemographic factors, healthcare utilization in the previous year, history of physical and neuropsychiatric comorbidities, and use of medications other than anticholinergics

^†^ *p* < 0.01

^‡^ *p* < 0.001

### Sensitivity analyses

Analysis using the entire control sample yielded similar results (Table S10). When using the Swedish anticholinergic burden scale, significant associations were observed for both strong and weak anticholinergic drugs (Table S11). Consistent results were obtained when implementing alternative covariate adjustments (Table S12). Inclusion of prevalent users of strong anticholinergic drugs modestly attenuated the association with these medications (Table S13).

## Discussion

This study found that use of anticholinergic drugs was associated with an increased risk of dementia, dependent on anticholinergic potency, cumulative dose, and drug class. A noticeable dose-response relationship was observed with strong anticholinergic drugs, while a subtle association lacking a dose-response pattern was noted for weak anticholinergic drugs. Among strong anticholinergic drugs, a significant association was found with urinary antispasmodics, antihistamines, and psychotropic drugs, but not with gastrointestinal drugs or muscle relaxants; a stronger association was observed in men, younger people, those diagnosed with vascular dementia or Lewy body dementia compared to Alzheimer’s disease, and those with milder-stage dementia.

Our finding of potency-dependent, dose-response associations, independent of certain drugs known to negatively impact cognition (e.g., opioids and benzodiazepines), supports a mechanistic link between anticholinergic burden and dementia. A few previous studies have similarly demonstrated a dose-response gradient for strong anticholinergics [10–12, 24], with the magnitude of effect sizes comparable to ours, contrasting with the absence of such a pattern for weak anticholinergics [11, 24]. Cumulative use of drugs with an ACB score of 1 contributes trivially, if any, to dementia risk. These medications exhibit affinity to muscarinic receptors in laboratory assays, but may not reliably predict adverse cognitive effects due to neglecting blood-brain barrier permeability [25]. Our analysis revealed a nonlinear dose-response curve with a ceiling effect for strong anticholinergic drugs, compatible with the hypothesis that receptor-mediated action will plateau at higher loads given the finite muscarinic receptors in the body [26]. Of note, prior research typically assessed anticholinergic burden by aggregating the scores of each dispensed drug, without considering cumulative dose [6–9]. This approach improperly assumes that drugs’ anticholinergic potency is proportional to their assigned points [27], thereby limiting its interpretability.

The drug class-specific associations might be explained by the distinct pharmacological properties of anticholinergics. A drug’s physicochemical and pharmacokinetic properties, and in particular its ability to cross the blood-brain barrier, are important determinants of central nervous system effects [28]. Furthermore, anticholinergic drugs can exhibit varying degrees of antagonism for different muscarinic receptor subtypes, with the M1 receptor playing a key role in cognitive function, while perhaps affecting other pathways [29, 30]. Existing literature has consistently linked the cumulative use of strong anticholinergic antidepressants, anti-Parkinson drugs, and urinary antispasmodics to a higher risk of dementia [9]. In the current study, significant associations were also observed with antihistamines and several other psychotropic drug classes (i.e., analgesics, antiepileptics, antipsychotics, and anxiolytics), partly owing to a large sample size. Additionally, the slightly negative associations with certain weak anticholinergics could suggest the involvement of mechanisms exerting opposite impacts on cognition. Alternatively, the varied associations across drug classes may imply the presence of protopathic bias and confounding by indication. For instance, anticholinergics, particularly those affecting the central nervous system, may have been prescribed for prodromal symptoms of dementia such as depression and psychosis that can manifest several years before a clinical diagnosis [31]. Moreover, neuropsychiatric conditions themselves can serve as a predictor of future dementia [32, 33]. Nevertheless, our study, along with others [11, 12, 34], yielded consistent results across different lengths of latency.

In line with previous research [12, 24], we found a more pronounced association in men and younger individuals. Older adults are deemed more susceptible to anticholinergic side effects due to decreased drug metabolism and clearance and increased permeability of the blood-brain barrier [2]. However, it should be noted that our analysis utilized relative effect measures, and thus, the lower odds ratio observed in older people could be attributed to their substantially higher age-related risk of dementia. In the older age group, death may more frequently occur before dementia diagnosis; this survival bias, owing to the depletion of more vulnerable individuals, could also attenuate the observed association. The reasons behind such sex differences remain unclear but may span multiple domains, including drug prescribing patterns, disease pathophysiology, and clinical phenotypes. The composition of anticholinergic drug burden varies by sex due to differences in underlying indications. For instance, a larger proportion of the burden may be attributed to antidepressants and urinary antispasmodics in women, in contrast to anti-Parkinson drugs and antipsychotics in men. One study reported a higher risk of dementia associated with urinary antispasmodic use in men than women [35], pointing to a need to clarify sex-specific risks for other classes of anticholinergic drugs. From a biological perspective, sex-specific variation in cholinergic function has been proposed, with females exhibiting higher cholinergic activity in the frontal cortex and males in the hippocampus [36]. Additionally, sex-dependent cholinergic effects on amyloid pathology, potentially mediated by estradiol, have been suggested [37]. Lastly, women tend to be older, have lower cognitive function at diagnosis, and exhibit different distributions of dementia subtypes, all of which may partially explain the observed sex differences.

The relationship between anticholinergic burden and dementia varied by disease subtype and severity, which has seldom been investigated. Intriguingly, compared with Alzheimer’s disease, we found a stronger association with vascular dementia, consistent with a previous study [12], but contradicted by another [38]. A neuroimaging study linked anticholinergic use to reduced brain glucose metabolism and increased cerebral atrophy, reflecting cholinergic neurodegeneration and dysfunction [39]. In addition, anticholinergics have been associated with elevated risks of myocardial infarction and ischemic stroke [40, 41], which are stronger predictors for vascular dementia than Alzheimer’s disease [42]. Potential vascular pathology involving the non-neuronal cholinergic system might be mediated by suppressed parasympathetic activity in the heart [43] and impaired cholinergic modulation of inflammation [44]. Our finding of the strongest association with Lewy body dementia is novel and warrants confirmation. The risk of protopathic bias may be higher in this subtype, which tends to manifest more and broader prodromal (e.g., neuropsychiatric, motor, and autonomic) symptoms [45]. Applying longer lag periods resulted in greater attenuation of the ORs for Lewy body dementia; however, it remained the subtype with the strongest association. One plausible explanation is that Lewy body dementia involves greater cholinergic deficits. Research indicates that cholinergic loss occurs earlier, is more widespread, and is spatially distinct in Lewy body dementia than in Alzheimer’s disease [46, 47], highlighting a heterogeneous role of the cholinergic system in neurodegenerative disorders.

Moreover, our study found that the use of strong anticholinergic drugs was more predictive of less severe dementia. We propose several possible explanations: (1) individuals with a lower MMSE at the time of registration in SveDem may have experienced longer diagnostic delays, which could blur the association; (2) those with a lower MMSE were likely at a later stage of disease, with greater loss of cholinergic innervation [3], where anticholinergic effects may be diminished; and (3) there may exist a ceiling effect, whereby the detrimental impact of anticholinergic drugs plateaus. In advanced stages of dementia, underlying pathologies such as amyloid-β and tau accumulation or vascular damage may become the primary drivers of cognitive decline [48]. This finding suggests that anticholinergic burden may represent a more relevant and modifiable risk factor at the earlier stages of dementia, underscoring the importance of minimizing unnecessary anticholinergic use, especially in younger individuals or those with milder cognitive symptoms.

There is an increasing recognition of the importance of reducing anticholinergic burden in older people [49]. However, evidence from a few randomized controlled trials of deprescribing interventions targeting overall anticholinergic burden demonstrates no clear impact on cognition and quality of life [50, 51]. It is noteworthy that interventions implemented in these trials did not effectively alleviate participants’ anticholinergic burden, partly because of short follow-up time and various barriers to deprescribing. Instead, future trials may concentrate on specific classes (e.g., urinary antispasmodics) or particular agents (e.g., hydroxyzine and amitriptyline) of anticholinergics, which contribute significantly to the overall burden and have alternative treatment options. Importantly, clinicians should remain vigilant when prescribing medications with strong anticholinergic properties to middle-aged and older adults, and whenever possible, consider alternative treatments or dose adjustments to minimize the potential adverse effects. Shared decision-making in prescribing and deprescribing, which involves choosing treatments based on both evidence and patients’ preferences, is essential to optimize treatment outcomes.

Strengths of this study include a large, representative study population across Sweden, with detailed information on prescriptions, confounders (including education and income), and subtype and severity of dementia. Several limitations should be acknowledged. First, given this study’s observational nature, a causal relationship between anticholinergic burden and dementia risk cannot be firmly established. The observed associations may be partially due to unmeasured confounders, indication bias, and protopathic bias, notwithstanding our efforts to control them. Future clinical trials on deprescribing anticholinergics will indirectly provide evidence for causality. Second, we lacked information on lifestyle factors such as smoking or alcohol use. Additionally, primary care data were not available, leading to the underdiagnosis of certain comorbidities such as depression. Third, the assessment of anticholinergic burden lacks consensus, and exposure misclassification is likely inevitable. We primarily relied on the ACB scale, a validated and widely used tool, to facilitate comparability with previous research. Although studies that applied and compared different scales generally report consistent positive associations [11, 34], the choice of scale is both region- and time-sensitive and may influence effect estimates. Individuals may not strictly adhere to their prescribed medication regimen, potentially underestimating the true effects. Our register data did not capture anticholinergics sold over the counter or those used in hospitals. Fourth, we cannot rule out the possibility of under-ascertainment of dementia and misclassification of its subtype and severity. Finally, generalizing these findings to other settings should be approached with caution, as prescribing and diagnostic practices vary across healthcare systems.

In conclusion, this nationwide case-control study reveals a dose-response relationship between dementia risk and cumulative use of strong anticholinergic drugs, particularly urinary antispasmodics, antihistamines, and psychotropic drugs, but no such pattern for weak anticholinergic drugs. The associations with strong anticholinergics were more pronounced in men and younger individuals, stronger for vascular dementia and Lewy body dementia compared to Alzheimer’s disease, and for milder-stage dementia.

## Supplementary Information

Supplementary Material 1.

## Data Availability

Requests for access to the SveDem data should be addressed to the registry holder and the steering committee (www.svedem.se).
