# Supplementary material for: Anticholinergic burden and incident dementia: a Swedish nationwide case-control study
Source: Alzheimers Res Ther. 2025 Oct 21;17:227. doi: 10.1186/s13195-025-01883-8 (PMC12538848; doi:10.1186/s13195-025-01883-8)
Supplement: Supplementary file 1 — Supplementary Material 1. [file 13195_2025_1883_MOESM1_ESM.pdf]

Supplementary Material

Table S1. List of ICD-10 and ATC codes used to identify dementia and its subtypes 2

Table S2. List of drugs included in the Anticholinergic Cognitive Burden scale 3

Table S3. List of ATC codes used to categorize drug class of anticholinergics 6

Table S4. List of ICD-10 and ATC codes used to define covariates.. 7

Table S5. Association between cumulative use of anticholinergic drugs and all-cause dementia, by sex.... 9

Table S6. Association between cumulative use of anticholinergic drugs and all-cause dementia, by age at diagnosis 10

Table S7. Association between cumulative use of specific classes of weak anticholinergic drugs and all-cause dementia 12

Table S8. Association between cumulative use of weak anticholinergic drugs and type-specific dementia 14

Table S9. Association between cumulative use of weak anticholinergic drugs and all-cause dementia, by MMSE score at diagnosis 15

Table S10. Association between cumulative use of anticholinergic drugs and all-cause dementia, using the entire control sample 16

Table S11. Association between cumulative use of anticholinergic drugs and all-cause dementia, using the Swedish anticholinergic burden scale 17

Table S12. Association between cumulative use of anticholinergic drugs and all-cause dementia, using alternative covariates adjustments 18

Table S13. Association between cumulative use of anticholinergic drugs and all-cause dementia, by incorporating prevalent users 19

Figure S1. Graphical illustration of the study design 20

Figure S2. Flowchart for the selection of dementia cases and matched controls 21

Figure S3. Number of prescriptions during the drug exposure period, by drug class 22

Figure S4. Number of defined daily doses during the drug exposure period, by drug class 23

Figure S5. Number of prescriptions during the drug exposure period, by individual drug......................... 24

Figure S6. Number of defined daily doses during the drug exposure period, by individual drug.............. 25

Figure S7. Association between cumulative use of weak anticholinergic drugs and all-cause dementia using restricted cubic splines 26

Table S1. List of ICD-10 and ATC codes used to identify dementia and its subtypes

	ICD-10 codes	ATC codes
**All-cause dementia**	F00-F03, G30-G31	N06DA02, N06DA03, N06DA04, N06DX01
**Type-specific dementia**		
Alzheimer's disease/ Mixed dementia	F00, G30	
Vascular dementia	F01	
Lewy body dementia*	F023, G318	
Frontotemporal dementia	G310	
Unspecified dementia	F03, G319	
Other dementias†	F020, F021, F022, F024, F028, G311, G312	

Abbreviations: ATC, Anatomical Therapeutic Chemical; ICD-10, International Classification of Diseases, Tenth Revision.

* Lewy body dementia includes dementia with Lewy bodies and Parkinson's disease dementia.

† Other miscellaneous types, such as cortico-basal syndrome or alcohol-related dementia.

Table S2. List of drugs included in the Anticholinergic Cognitive Burden scale

Generic name	Score	ATC codes
Alimemazine	1	R06AD01
Alprazolam	1	N05BA12
Alverine	1	A03AX08, A03AX58
Aripiprazole	1	N05AX12
Asenapine	1	N05AH05
Atenolol	1	C07AB03, C07AB11, C07BB03, C07CB03, C07CB53, C07DB01, C07FB03
Bupropion	1	N06AX12, A08AA62
Captopril	1	C09AA01, C09BA01
Cetirizine	1	R06AE07
Chlortalidone	1	C03BA04, C03BB04, C03EA06
Cimetidine	1	A02BA01, A02BA51
Clidinium	1	A03CA02
Clorazepate	1	N05BA05
Codeine	1	R05DA04, R05DA12, N02AA08, N02AA58, N02AA59, N02AA79, N02AJ01, N02AJ02, N02AJ03, N02AJ06, N02AJ07, N02AJ08, N02AJ09
Colchicine	1	M04AC01, L01CC
Desloratadine	1	R06AX27
Diazepam	1	N05BA01, N05BA17
Digoxin	1	C01AA05, C01AA02, C01AA08, C01AA52
Dipyridamole	1	B01AC07
Disopyramide	1	C01BA03
Fentanyl	1	N01AH01, N01AH51, N02AB03
Fluvoxamine	1	N06AB08
Furosemide	1	C03CA01, C03CB01, C03EB01
Haloperidol	1	N05AD01
Hydralazine	1	C02DB02, C02DB01, C02LG01, C02LG02, C02LG51
Hydrocortisone	1	H02AB09, A07EA02
Iloperidone	1	N05AX14
Isosorbide	1	C01DA08, C01DA14, C01DA58, C05AE02
Levocetirizine	1	R06AE09
Loperamide	1	A07DA03, A07DA05, A07DA53
Loratadine	1	R06AX13
Metoprolol	1	C07AB02, C07BB02, C07BB52, C07CB02, C07FB02, C07FB13, C07FX03, C07FX05

Generic name	Score	ATC codes
Morphine	1	N02AA01, N02AA04, N02AA51, N02AG01, R05DA01, R05DA05, A07DA52
Nifedipine	1	C08CA05, C08CA55, C08GA01
Paliperidone	1	N05AX13
Prednisone	1	H02AB07, H02AB15, A07EA03
Quinidine	1	C01BA01, C01BA13, C01BA51, C01BA71
Ranitidine	1	A02BA02, A02BA07
Risperidone	1	N05AX08
Theophylline	1	R03DA04, R03DA54, R03DA74, R03DB04
Trazodone	1	N06AX05
Triamterene	1	C03DB02
Venlafaxine	1	N06AX16
Warfarin	1	B01AA03
Amantadine	2	N04BB01
Belladonna	2	A03BA04, A03CB02, A06AB30
Carbamazepine	2	N03AF01
Cyclobenzaprine	2	M03BX08
Cyproheptadine	2	R06AX02
Levomepromazine	2	N05AA02
Loxapine	2	N05AH01
Molindone	2	N05AE02
Nefopam	2	N02BG06
Oxcarbazepine	2	N03AF02
Pethidine	2	N02AB02, N02AB52, N02AB72, N02AG03
Pimozide	2	N05AG02
Amitriptyline	3	N06AA09, N06CA01
Amoxapine	3	N06AA17
Atropine	3	A03BA01, A03CB03, A03BB02, A03BB06, A03CB04
Benzatropine	3	N04AC01, N04AC30
Brompheniramine	3	R06AB01, R06AB51, R06AB06, R06AB56
Carbinoxamine	3	R06AA08
Chlorphenamine	3	R06AB04, R06AB54, R06AB02, R06AB52
Chlorpromazine	3	N05AA01
Clemastine	3	R06AA04, R06AA54
Clomipramine	3	N06AA04

Generic name	Score	ATC codes
Clozapine	3	N05AH02
Darifenacin	3	G04BD10
Desipramine	3	N06AA01
Dicycloverine	3	A03AA07
Dimenhydrinate	3	R06AA11, R06AA61
Diphenhydramine	3	R06AA02, R06AA52
Doxepin	3	N06AA12
Doxylamine	3	R06AA09, R06AA59
Fesoterodine	3	G04BD11, G04BD13
Flavoxate	3	G04BD02
Hydroxyzine	3	N05BB01, N05BB51
Hyoscyamine	3	A03BA03, A03CB31
Imipramine	3	N06AA02, N06AA03
Meclozine	3	R06AE05, R06AE55
Methocarbamol	3	M03BA03, M03BA53, M03BA73
Nortriptyline	3	N06AA10
Olanzapine	3	N05AH03, N05AH53
Orphenadrine	3	M03BC01, M03BC51, N04AB02
Oxybutynin	3	G04BD04
Paroxetine	3	N06AB05
Perphenazine	3	N05AB03
Promethazine	3	R06AD02, R06AD52, V03AB05, R06AD05, R06AD55
Propantheline	3	A03AB05, A03CA34
Propiverine	3	G04BD06
Quetiapine	3	N05AH04
Scopolamine	3	A04AD01, A04AD51, N05CM05, A03BB01, A03BB03, A03CB01, A03DB04
Solifenacin	3	G04BD08, G04CA53
Thioridazine	3	N05AC02
Tolterodine	3	G04BD07
Trifluoperazine	3	N05AB06
Trihexyphenidyl	3	N04AA01
Trimipramine	3	N06AA06
Trospium	3	G04BD09, A03DA06

Abbreviation: ATC, Anatomical Therapeutic Chemical.

Table S3. List of ATC codes used to categorize drug class of anticholinergics

Drug class	ATC code
Gastrointestinal drugs	A
Antithrombotic drugs	B01A
Cardiovascular drugs	C
Urinary antispasmodics	G04BD
Glucocorticoids	H02AB
Musculoskeletal system drugs	M
Antihistamines	R06A
Analgesics	N02
Antiepileptics	N03A
Anti-Parkinson drugs	N04
Antipsychotics	N05A
Anxiolytics	N05B
Antidepressants	N06A

Table S4. List of ICD-10 and ATC codes used to define covariates

	ICD-10 codes	ATC codes
**Physical comorbidities**		
Hypertension	I10-I15	C03, C07, C08, C09
Diabetes mellitus	E10-E14	A10A, A10B
Dyslipidemia	E78	
Myocardial infarction	I21, I22, I252	
Congestive heart failure	I110, I130, I132, I255, I420, I426-I429, I43, I50	
Peripheral vascular disease	I70, I71, I731, I738, I739, I771, I790, I792, K55	
Atrial fibrillation	I48	
Stroke	G45, I60-I64, I67, I69	
Hemiplegia/Tetraplegia	G114, G80-G82, G830-G833, G838	
Any cancer (excl. non-melanoma skin cancer)	C00-C97, excl. C44	
Rheumatic disease	M05, M06, M123, M070-M073, M08, M13, M30, M313-M316, M32-M34, M350, M351, M353, M45, M46	
Osteoarthritis	M15-M19	
Liver disease	B15-B19, K70-K77, R18, I850, I859, I982, I983	
Lung disease	J41-J47, J60-J70	
Kidney disease	N032-N037, N052-N057, N11, N18, N19, N250, I120, I131, Q611, Q614, Z49, Z940, Z992	
Urinary incontinence	N393, N394, R32	
Gastro-esophageal reflux disease/Esophagitis	K20, K21	
Peptic ulcer disease	K25-K28	
Inflammatory bowel disease	K50, K51, K523	
Irritable bowel syndrome	K58	
Hearing loss	H833, H90, H91	
Vestibular disorders	H81	
Back pain	M54	
Chronic pain	F454, E104, E114, E124, E134, E144, B022, G500, G501, G530, M255, M501, M511, M541, M791, M792, M797, G631, G632, R521, R522	

	ICD-10 codes	ATC codes
**Neuropsychiatric comorbidities**		
Parkinson's disease	G20	
Epilepsy	G40	
Migraine/Headache	G43, G44, R51	
Substance use disorder	F10-F19	
Psychotic disorders/Bipolar disorder	F20-F29, F30, F31	
Depression	F32, F33	
Anxiety disorders	F40, F41	
Stress-related disorders	F43	
Sleep disorders	F51, G47	
**Medications**		
Renin-angiotensin system inhibitors		C09
Beta-blockers		C07
Calcium channel blockers		C08
Diuretics		C03
Statins		C10AA, C10B
Proton-pump inhibitors		A02BC
Antiplatelet drugs		B01AC
Nonsteroidal anti-inflammatory drugs		M01A
Antihistamines		R06A
Antispasmodics		A03, G04BD
Analgesics		N02
Antiepileptics		N03A
Anti-Parkinson drugs		N04
Antipsychotics		N05A
Anxiolytics, hypnotics, and sedatives		N05B, N05C
Antidepressants		N06A

Abbreviations: ATC, Anatomical Therapeutic Chemical; ICD-10, International Classification of Diseases, Tenth Revision.

Table S5. Association between cumulative use of anticholinergic drugs and all-cause dementia, by sex

Exposure, DDDs	No. of cases (%)	No. of controls (%)	Odds ratio (95% CI)*		
			Model 1	Model 2	Model 3
**Men**					
Strong anticholinergics					
0	66,324 (79.7)	70,789 (85.1)	1.00	1.00	1.00
1-89	9383 (11.3)	7978 (9.6)	1.24 (1.20, 1.28)‡	1.22 (1.18, 1.26)‡	1.21 (1.17, 1.25)‡
90-364	4458 (5.4)	2879 (3.5)	1.63 (1.55, 1.71)‡	1.55 (1.47, 1.62)‡	1.51 (1.43, 1.59)‡
365-1094	2068 (2.5)	1112 (1.3)	1.96 (1.82, 2.11)‡	1.80 (1.67, 1.94)‡	1.74 (1.61, 1.88)‡
≥1095	972 (1.2)	447 (0.5)	2.31 (2.07, 2.59)‡	2.07 (1.85, 2.33)‡	2.01 (1.78, 2.26)‡
Weak anticholinergics					
0	26,190 (31.5)	28,885 (34.7)	1.00	1.00	1.00
1-89	8014 (9.6)	7586 (9.1)	1.13 (1.09, 1.17)‡	1.12 (1.08, 1.16)‡	1.11 (1.07, 1.15)‡
90-364	9457 (11.4)	8600 (10.3)	1.17 (1.13, 1.21)‡	1.15 (1.11, 1.19)‡	1.14 (1.10, 1.18)‡
365-1094	14,099 (16.9)	13,297 (16.0)	1.13 (1.10, 1.17)‡	1.10 (1.07, 1.14)‡	1.09 (1.05, 1.13)‡
≥1095	25,445 (30.6)	24,837 (29.9)	1.09 (1.06, 1.12)‡	1.04 (1.01, 1.07)†	1.04 (1.01, 1.07)
**Women**					
Strong anticholinergics					
0	90,779 (78.0)	93,982 (80.8)	1.00	1.00	1.00
1-89	15,473 (13.3)	15,183 (13.1)	1.05 (1.03, 1.08)‡	1.04 (1.02, 1.07)†	1.04 (1.01, 1.07)†
90-364	6215 (5.3)	4659 (4.0)	1.38 (1.33, 1.44)‡	1.34 (1.29, 1.40)‡	1.31 (1.25, 1.36)‡
365-1094	2695 (2.3)	1768 (1.5)	1.59 (1.50, 1.69)‡	1.50 (1.41, 1.60)‡	1.45 (1.36, 1.55)‡
≥1095	1159 (1.0)	729 (0.6)	1.66 (1.51, 1.82)‡	1.52 (1.39, 1.68)‡	1.45 (1.32, 1.60)‡
Weak anticholinergics					
0	33,035 (28.4)	34,499 (29.7)	1.00	1.00	1.00
1-89	13,222 (11.4)	12,122 (10.4)	1.12 (1.09, 1.15)‡	1.11 (1.08, 1.15)‡	1.10 (1.06, 1.13)‡
90-364	14,089 (12.1)	12,963 (11.1)	1.11 (1.08, 1.14)‡	1.11 (1.07, 1.14)‡	1.09 (1.06, 1.12)‡
365-1094	20,210 (17.4)	19,665 (16.9)	1.05 (1.02, 1.08)‡	1.05 (1.02, 1.08)‡	1.04 (1.01, 1.07)†
≥1095	35,765 (30.7)	37,072 (31.9)	0.98 (0.96, 1.00)	0.97 (0.95, 1.00)	0.98 (0.95, 1.01)

Abbreviations: CI, confidence interval; DDD, defined daily dose.

* Model 1 was unadjusted; Model 2 was adjusted for sociodemographic factors, healthcare utilization in the previous year, and history of physical and neuropsychiatric comorbidities; Model 3 was additionally adjusted for the use of other medications.

† p <0.01

‡ p <0.001

Table S6. Association between cumulative use of anticholinergic drugs and all-cause dementia, by age at diagnosis

Exposure, DDDs	No. of cases (%)	No. of controls (%)	Odds ratio (95% CI)*		
			Model 1	Model 2	Model 3
**<65 years**					
Strong anticholinergics					
0	5185 (72.2)	6163 (85.7)	1.00	1.00	1.00
1-89	1047 (14.6)	725 (10.1)	1.59 (1.43, 1.77)‡	1.48 (1.31, 1.66)‡	1.47 (1.30, 1.66)‡
90-364	565 (7.9)	206 (2.9)	2.87 (2.40, 3.41)‡	2.42 (2.00, 2.93)‡	2.32 (1.91, 2.81)‡
365-1094	242 (3.4)	66 (0.9)	3.96 (2.95, 5.32)‡	3.21 (2.33, 4.43)‡	3.07 (2.21, 4.25)‡
≥1095	144 (2.0)	28 (0.4)	5.51 (3.55, 8.57)‡	3.72 (2.33, 5.95)‡	3.47 (2.15, 5.58)‡
Weak anticholinergics					
0	3357 (46.7)	4421 (61.5)	1.00	1.00	1.00
1-89	1106 (15.4)	999 (13.9)	1.33 (1.20, 1.48)‡	1.30 (1.16, 1.45)‡	1.29 (1.15, 1.45)‡
90-364	937 (13.0)	658 (9.2)	1.63 (1.45, 1.83)‡	1.50 (1.32, 1.71)‡	1.50 (1.32, 1.72)‡
365-1094	839 (11.7)	538 (7.5)	1.78 (1.57, 2.02)‡	1.58 (1.37, 1.83)‡	1.59 (1.36, 1.85)‡
≥1095	944 (13.1)	572 (8.0)	1.83 (1.61, 2.07)‡	1.42 (1.22, 1.65)‡	1.43 (1.22, 1.69)‡
**65-74 years**					
Strong anticholinergics					
0	20,974 (76.6)	23,372 (85.2)	1.00	1.00	1.00
1-89	3537 (12.9)	2772 (10.1)	1.38 (1.31, 1.46)‡	1.32 (1.24, 1.40)‡	1.30 (1.23, 1.38)‡
90-364	1617 (5.9)	792 (2.9)	2.16 (1.97, 2.37)‡	1.93 (1.75, 2.12)‡	1.86 (1.69, 2.05)‡
365-1094	784 (2.9)	331 (1.2)	2.56 (2.24, 2.93)‡	2.07 (1.80, 2.39)‡	2.01 (1.75, 2.32)‡
≥1095	453 (1.7)	151 (0.6)	3.18 (2.63, 3.86)‡	2.46 (2.02, 3.01)‡	2.26 (1.85, 2.76)‡
Weak anticholinergics					
0	10,813 (39.5)	13,040 (47.6)	1.00	1.00	1.00
1-89	3421 (12.5)	3421 (12.5)	1.16 (1.10, 1.23)‡	1.12 (1.06, 1.19)‡	1.11 (1.05, 1.18)‡
90-364	3270 (11.9)	2879 (10.5)	1.30 (1.22, 1.37)‡	1.22 (1.14, 1.30)‡	1.21 (1.14, 1.29)‡
365-1094	3939 (14.4)	3445 (12.6)	1.29 (1.22, 1.37)‡	1.15 (1.09, 1.23)‡	1.15 (1.08, 1.23)‡
≥1095	5922 (21.6)	4633 (16.9)	1.43 (1.36, 1.50)‡	1.17 (1.10, 1.24)‡	1.18 (1.10, 1.26)‡
**75-84 years**					
Strong anticholinergics					
0	62,205 (78.1)	65,673 (82.5)	1.00	1.00	1.00
1-89	10,142 (12.7)	9253 (11.6)	1.16 (1.12, 1.20)‡	1.14 (1.10, 1.18)‡	1.13 (1.10, 1.17)‡

Exposure, DDDs	No. of cases (%)	No. of controls (%)	Odds ratio (95% CI)*		
			Model 1	Model 2	Model 3
90-364	4439 (5.6)	3039 (3.8)	1.55 (1.48, 1.63)‡	1.50 (1.43, 1.57)‡	1.46 (1.39, 1.54)‡
365-1094	1983 (2.5)	1192 (1.5)	1.78 (1.65, 1.92)‡	1.68 (1.56, 1.82)‡	1.63 (1.51, 1.76)‡
≥1095	916 (1.1)	487 (0.6)	1.98 (1.77, 2.21)‡	1.84 (1.64, 2.06)‡	1.79 (1.60, 2.01)‡
Weak anticholinergics					
0	24,823 (31.2)	26,400 (33.1)	1.00	1.00	1.00
1-89	8421 (10.6)	8197 (10.3)	1.06 (1.02, 1.10)†	1.05 (1.01, 1.09)	1.04 (1.00, 1.08)
90-364	9474 (11.9)	8962 (11.3)	1.08 (1.05, 1.12)‡	1.07 (1.03, 1.11)‡	1.06 (1.02, 1.10)†
365-1094	13,611 (17.1)	13,295 (16.7)	1.05 (1.02, 1.09)‡	1.04 (1.00, 1.07)	1.03 (0.99, 1.06)
≥1095	23,356 (29.3)	22,790 (28.6)	1.05 (1.03, 1.08)‡	1.00 (0.97, 1.04)	1.01 (0.97, 1.04)
**≥85 years**					
Strong anticholinergics					
0	68,739 (80.6)	69,563 (81.6)	1.00	1.00	1.00
1-89	10,130 (11.9)	10,411 (12.2)	0.99 (0.96, 1.02)	0.99 (0.96, 1.02)	0.99 (0.96, 1.02)
90-364	4052 (4.8)	3501 (4.1)	1.18 (1.13, 1.24)‡	1.17 (1.12, 1.23)‡	1.15 (1.10, 1.21)‡
365-1094	1754 (2.1)	1291 (1.5)	1.39 (1.29, 1.50)‡	1.36 (1.27, 1.47)‡	1.32 (1.22, 1.42)‡
≥1095	618 (0.7)	510 (0.6)	1.24 (1.10, 1.40)‡	1.23 (1.09, 1.39)‡	1.19 (1.06, 1.35)†
Weak anticholinergics					
0	20,232 (23.7)	19,523 (22.9)	1.00	1.00	1.00
1-89	8288 (9.7)	7091 (8.3)	1.12 (1.08, 1.16)‡	1.12 (1.08, 1.17)‡	1.10 (1.06, 1.14)‡
90-364	9865 (11.6)	9064 (10.6)	1.04 (1.00, 1.07)	1.06 (1.02, 1.10)†	1.04 (1.01, 1.08)
365-1094	15,920 (18.7)	15,684 (18.4)	0.97 (0.94, 1.00)	1.01 (0.98, 1.04)	1.00 (0.96, 1.03)
≥1095	30,988 (36.3)	33,914 (39.8)	0.87 (0.85, 0.89)‡	0.91 (0.89, 0.94)‡	0.92 (0.89, 0.95)‡

Abbreviations: CI, confidence interval; DDD, defined daily dose.

* Model 1 was unadjusted; Model 2 was adjusted for sociodemographic factors, healthcare utilization in the previous year, and history of physical and neuropsychiatric comorbidities; Model 3 was additionally adjusted for the use of other medications.

† p <0.01

‡ p <0.001

Table S7. Association between cumulative use of specific classes of weak anticholinergic drugs and all-cause dementia

Exposure, DDDs	No. of cases (%)	No. of controls (%)	Adjusted odds ratio (95% CI)[*]		
			1-year lag	3-year lag	5-year lag
**Gastrointestinal drugs**					
0	185,652 (93.0)	185,721 (93.1)	1.00	1.00	1.00
1-89	8849 (4.4)	8585 (4.3)	0.97 (0.94, 1.00)	0.99 (0.95, 1.03)	0.99 (0.94, 1.04)
90-364	3238 (1.6)	3309 (1.7)	0.93 (0.88, 0.98)[†]	0.95 (0.89, 1.00)	0.98 (0.91, 1.05)
≥365	1787 (0.9)	1911 (1.0)	0.88 (0.82, 0.94)[‡]	0.90 (0.82, 0.97)[†]	0.87 (0.78, 0.97)
**Antithrombotic drugs**					
0	169,008 (84.7)	169,635 (85.0)	1.00	1.00	1.00
1-89	2934 (1.5)	2853 (1.4)	1.04 (0.98, 1.09)	1.08 (1.02, 1.15)	1.14 (1.05, 1.23)[†]
90-364	8493 (4.3)	8508 (4.3)	1.02 (0.98, 1.05)	1.07 (1.03, 1.11)[‡]	1.10 (1.05, 1.15)[‡]
≥365	19,091 (9.6)	18,530 (9.3)	1.04 (1.02, 1.07)[‡]	1.09 (1.06, 1.12)[‡]	1.08 (1.04, 1.13)[‡]
**Cardiovascular drugs**					
0	92,236 (46.2)	91,543 (45.9)	1.00	1.00	1.00
1-89	8542 (4.3)	8343 (4.2)	0.98 (0.95, 1.01)	0.98 (0.95, 1.02)	1.01 (0.96, 1.05)
90-364	18,423 (9.2)	17,448 (8.7)	1.01 (0.98, 1.03)	0.99 (0.96, 1.01)	0.99 (0.96, 1.02)
≥365	80,325 (40.3)	82,192 (41.2)	0.93 (0.91, 0.95)[‡]	0.95 (0.93, 0.97)[‡]	0.96 (0.94, 0.99)[†]
**Glucocorticoids**					
0	198,698 (99.6)	198,662 (99.6)	1.00	1.00	1.00
1-89	47 (<0.1)	45 (<0.1)	0.99 (0.64, 1.53)	0.97 (0.56, 1.69)	0.76 (0.36, 1.60)
90-364	368 (0.2)	453 (0.2)	0.83 (0.72, 0.96)	1.01 (0.84, 1.20)	1.09 (0.87, 1.36)
≥365	413 (0.2)	366 (0.2)	1.15 (1.00, 1.33)	1.22 (1.01, 1.47)	1.15 (0.90, 1.48)
**Musculoskeletal system drugs**					
0	199,154 (99.8)	199,071 (99.8)	1.00	1.00	1.00
1-89	278 (0.1)	335 (0.2)	0.89 (0.75, 1.05)	0.79 (0.63, 0.97)	1.08 (0.80, 1.46)
90-364	73 (<0.1)	92 (<0.1)	0.78 (0.57, 1.07)	0.84 (0.57, 1.24)	0.92 (0.55, 1.55)
≥365	21 (<0.1)	28 (<0.1)	0.78 (0.43, 1.39)	0.94 (0.46, 1.94)	1.36 (0.43, 4.36)
**Antihistamines**					
0	181,320 (90.9)	181,474 (91.0)	1.00	1.00	1.00
1-89	7593 (3.8)	7416 (3.7)	0.97 (0.94, 1.00)	1.00 (0.96, 1.05)	1.02 (0.97, 1.07)
90-364	6221 (3.1)	6164 (3.1)	0.94 (0.91, 0.98)[†]	0.98 (0.93, 1.02)	0.99 (0.94, 1.05)
≥365	4392 (2.2)	4472 (2.2)	0.90 (0.86, 0.95)[‡]	0.96 (0.90, 1.02)	0.96 (0.89, 1.04)

Exposure, DDDs	No. of cases (%)	No. of controls (%)	Adjusted odds ratio (95% CI)*		
			1-year lag	3-year lag	5-year lag
**Analgesics**					
0	160,897 (80.6)	162,862 (81.6)	1.00	1.00	1.00
1-89	29,077 (14.6)	28,621 (14.3)	1.00 (0.98, 1.02)	1.03 (1.01, 1.05)	1.04 (1.01, 1.08)†
90-364	5872 (2.9)	4967 (2.5)	1.09 (1.05, 1.14)‡	1.11 (1.06, 1.17)‡	1.11 (1.04, 1.19)†
≥365	3680 (1.8)	3076 (1.5)	1.06 (1.01, 1.12)	1.15 (1.08, 1.23)‡	1.17 (1.07, 1.28)‡
**Antipsychotics**					
0	188,535 (94.5)	197,537 (99.0)	1.00	1.00	1.00
1-89	8002 (4.0)	1380 (0.7)	5.37 (5.05, 5.70)‡	4.07 (3.72, 4.46)‡	2.91 (2.54, 3.33)‡
90-364	2444 (1.2)	437 (0.2)	4.58 (4.11, 5.10)‡	3.23 (2.79, 3.74)‡	2.31 (1.89, 2.83)‡
≥365	545 (0.3)	172 (0.1)	1.95 (1.62, 2.34)‡	1.59 (1.26, 2.01)‡	1.33 (0.97, 1.83)
**Anxiolytics**					
0	187,189 (93.8)	189,811 (95.1)	1.00	1.00	1.00
1-89	7186 (3.6)	5520 (2.8)	1.11 (1.07, 1.16)‡	1.09 (1.04, 1.14)‡	1.06 (1.00, 1.12)
90-364	2533 (1.3)	2220 (1.1)	0.97 (0.92, 1.03)	1.01 (0.95, 1.09)	1.00 (0.92, 1.09)
≥365	2618 (1.3)	1975 (1.0)	0.96 (0.90, 1.02)	0.97 (0.90, 1.05)	0.95 (0.87, 1.05)
**Antidepressants**					
0	194,783 (97.6)	197,204 (98.8)	1.00	1.00	1.00
1-89	1391 (0.7)	850 (0.4)	1.36 (1.24, 1.48)‡	1.29 (1.16, 1.43)‡	1.40 (1.23, 1.59)‡
90-364	1081 (0.5)	488 (0.2)	1.65 (1.47, 1.85)‡	1.55 (1.36, 1.78)‡	1.45 (1.22, 1.72)‡
≥365	2271 (1.1)	984 (0.5)	1.48 (1.36, 1.61)‡	1.44 (1.31, 1.58)‡	1.32 (1.17, 1.49)‡

Abbreviations: CI, confidence interval; DDD, defined daily dose.

Numbers of cases and controls are presented for the analysis using a 1-year lag time in the exposure assessment.

* Models were adjusted for sociodemographic factors, healthcare utilization in the previous year, history of physical and neuropsychiatric comorbidities, and use of medications other than anticholinergics.

† $p < 0.01$

‡ $p < 0.001$

Table S8. Association between cumulative use of weak anticholinergic drugs and type-specific dementia

Exposure, DDDs	No. of cases (%)	No. of controls (%)	Adjusted odds ratio (95% CI)*		
			1-year lag	3-year lag	5-year lag
**Alzheimer's disease/Mixed dementia**					
0	17,205 (37.8)	15,654 (34.4)	1.00	1.00	1.00
1-89	5571 (12.2)	5022 (11.0)	0.98 (0.94, 1.03)	0.98 (0.93, 1.03)	1.03 (0.97, 1.10)
90-364	5346 (11.8)	5072 (11.2)	0.93 (0.88, 0.97)†	0.90 (0.86, 0.95)‡	0.95 (0.90, 1.01)
≥365	17,357 (38.2)	19,731 (43.4)	0.79 (0.76, 0.82)‡	0.82 (0.78, 0.85)‡	0.85 (0.81, 0.90)‡
**Vascular dementia**					
0	5905 (23.5)	8208 (32.6)	1.00	1.00	1.00
1-89	2207 (8.8)	2614 (10.4)	1.10 (1.03, 1.18)†	1.12 (1.03, 1.20)†	1.08 (0.99, 1.18)
90-364	2905 (11.6)	2609 (10.4)	1.39 (1.30, 1.49)‡	1.29 (1.20, 1.39)‡	1.27 (1.17, 1.37)‡
≥365	14,125 (56.2)	11,711 (46.6)	1.38 (1.31, 1.45)‡	1.34 (1.26, 1.42)‡	1.30 (1.21, 1.40)‡
**Lewy body dementia**					
0	1597 (37.2)	1659 (38.6)	1.00	1.00	1.00
1-89	629 (14.7)	494 (11.5)	1.18 (1.00, 1.39)	1.17 (0.98, 1.40)	1.03 (0.84, 1.27)
90-364	572 (13.3)	462 (10.8)	1.10 (0.93, 1.31)	1.06 (0.88, 1.28)	1.15 (0.93, 1.42)
≥365	1495 (34.8)	1678 (39.1)	0.91 (0.78, 1.06)	0.89 (0.75, 1.05)	0.79 (0.65, 0.96)
**Frontotemporal dementia**					
0	731 (43.0)	789 (46.4)	1.00	1.00	1.00
1-89	245 (14.4)	214 (12.6)	1.12 (0.90, 1.40)	1.00 (0.78, 1.27)	0.90 (0.67, 1.21)
90-364	191 (11.2)	176 (10.4)	1.03 (0.80, 1.32)	0.94 (0.71, 1.23)	0.89 (0.65, 1.22)
≥365	532 (31.3)	520 (30.6)	0.97 (0.77, 1.21)	0.92 (0.71, 1.18)	1.19 (0.86, 1.63)

Abbreviations: CI, confidence interval; DDD, defined daily dose.

Analyses were performed among individuals with a specific type of dementia and their matched controls. Numbers of cases and controls are presented for the analysis using a 1-year lag time in the exposure assessment.

* Models were adjusted for sociodemographic factors, healthcare utilization in the previous year, history of physical and neuropsychiatric comorbidities, and use of medications other than anticholinergics.

† $p < 0.01$

‡ $p < 0.001$

Table S9. Association between cumulative use of weak anticholinergic drugs and all-cause dementia, by MMSE score at diagnosis

Exposure, DDDs	No. of cases (%)	No. of controls (%)	Adjusted odds ratio (95% CI)*		
			1-year lag	3-year lag	5-year lag
**MMSE score 25-30**					
0	5263 (36.0)	5406 (37.0)	1.00	1.00	1.00
1-89	1902 (13.0)	1667 (11.4)	1.12 (1.03, 1.21)†	1.09 (0.99, 1.19)	1.10 (0.99, 1.22)
90-364	1804 (12.3)	1562 (10.7)	1.12 (1.03, 1.22)†	1.07 (0.98, 1.17)	1.04 (0.93, 1.15)
≥365	5654 (38.7)	5988 (40.9)	0.98 (0.91, 1.05)	0.94 (0.86, 1.01)	0.94 (0.85, 1.04)
**MMSE score 20-24**					
0	7889 (33.5)	8044 (34.1)	1.00	1.00	1.00
1-89	2810 (11.9)	2608 (11.1)	1.04 (0.98, 1.11)	1.02 (0.96, 1.10)	1.04 (0.95, 1.12)
90-364	2670 (11.3)	2490 (10.6)	1.02 (0.95, 1.09)	1.03 (0.96, 1.10)	0.99 (0.92, 1.07)
≥365	10,214 (43.3)	10,441 (44.3)	0.90 (0.86, 0.95)‡	0.93 (0.87, 0.98)	0.94 (0.88, 1.01)
**MMSE score 10-19**					
0	6366 (33.5)	6122 (32.2)	1.00	1.00	1.00
1-89	2080 (10.9)	2029 (10.7)	0.98 (0.91, 1.06)	0.98 (0.91, 1.06)	1.07 (0.97, 1.17)
90-364	2089 (11.0)	2030 (10.7)	0.95 (0.89, 1.03)	0.93 (0.86, 1.01)	0.97 (0.89, 1.06)
≥365	8463 (44.5)	8817 (46.4)	0.87 (0.82, 0.93)‡	0.91 (0.86, 0.98)†	0.98 (0.91, 1.06)
**MMSE score 0-9**					
0	606 (38.0)	512 (32.1)	1.00	1.00	1.00
1-89	168 (10.5)	165 (10.3)	0.90 (0.68, 1.19)	0.77 (0.56, 1.06)	0.76 (0.51, 1.14)
90-364	172 (10.8)	185 (11.6)	0.82 (0.62, 1.07)	0.78 (0.58, 1.03)	0.86 (0.61, 1.20)
≥365	650 (40.7)	734 (46.0)	0.76 (0.61, 0.95)	0.85 (0.67, 1.09)	0.88 (0.65, 1.18)

Abbreviations: CI, confidence interval; DDD, defined daily dose; MMSE, Mini-Mental State Examination.

Analyses were performed among dementia patients with an available MMSE score and their matched controls. Numbers of cases and controls are presented for the analysis using a 1-year lag time in the exposure assessment.

* Models were adjusted for sociodemographic factors, healthcare utilization in the previous year, history of physical and neuropsychiatric comorbidities, and use of medications other than anticholinergics.

† $p < 0.01$

‡ $p < 0.001$

Table S10. Association between cumulative use of anticholinergic drugs and all-cause dementia, using the entire control sample

Exposure, DDDs	No. of cases (%)	No. of controls (%)	Odds ratio (95% CI)[*]		
			Model 1	Model 2	Model 3
Strong anticholinergics					
0	157,103 (78.7)	438,020 (82.7)	1.00	1.00	1.00
1-89	24,856 (12.5)	60,908 (11.5)	1.15 (1.13, 1.17)[‡]	1.13 (1.11, 1.15)[‡]	1.12 (1.10, 1.14)[‡]
90-364	10,673 (5.3)	19,606 (3.7)	1.54 (1.50, 1.58)[‡]	1.47 (1.43, 1.51)[‡]	1.44 (1.40, 1.48)[‡]
365-1094	4763 (2.4)	7567 (1.4)	1.82 (1.76, 1.89)[‡]	1.69 (1.62, 1.75)[‡]	1.62 (1.56, 1.69)[‡]
≥1095	2131 (1.1)	3414 (0.6)	1.95 (1.84, 2.06)[‡]	1.76 (1.66, 1.86)[‡]	1.67 (1.58, 1.77)[‡]
Weak anticholinergics					
0	59,225 (29.7)	183,558 (34.7)	1.00	1.00	1.00
1-89	21,236 (10.6)	55,757 (10.5)	1.12 (1.10, 1.14)[‡]	1.11 (1.09, 1.13)[‡]	1.10 (1.08, 1.12)[‡]
90-364	23,546 (11.8)	57,144 (10.8)	1.14 (1.12, 1.16)[‡]	1.12 (1.10, 1.15)[‡]	1.11 (1.09, 1.13)[‡]
365-1094	34,309 (17.2)	83,619 (15.8)	1.09 (1.07, 1.10)[‡]	1.07 (1.05, 1.09)[‡]	1.06 (1.04, 1.08)[‡]
≥1095	61,210 (30.7)	149,437 (28.2)	1.06 (1.05, 1.08)[‡]	1.02 (1.01, 1.04)[†]	1.03 (1.01, 1.05)[†]

Abbreviations: CI, confidence interval; DDD, defined daily dose.

[*] Model 1 was unadjusted; Model 2 was adjusted for sociodemographic factors, healthcare utilization in the previous year, and history of physical and neuropsychiatric comorbidities; Model 3 was additionally adjusted for the use of other medications.

[†] $p < 0.01$

[‡] $p < 0.001$

Table S11. Association between cumulative use of anticholinergic drugs and all-cause dementia, using the Swedish anticholinergic burden scale

Exposure, DDDs	No. of cases (%)	No. of controls (%)	Odds ratio (95% CI)*		
			Model 1	Model 2	Model 3
Strong anticholinergics					
0	106,697 (63.8)	112,832 (67.5)	1.00	1.00	1.00
1-89	36,947 (22.1)	36,765 (22.0)	1.03 (1.01, 1.05)†	1.03 (1.01, 1.04)†	1.03 (1.01, 1.05)†
90-364	14,202 (8.5)	11,396 (6.8)	1.24 (1.21, 1.28)‡	1.22 (1.19, 1.25)‡	1.22 (1.19, 1.25)‡
365-1094	6405 (3.8)	4306 (2.6)	1.48 (1.42, 1.54)‡	1.42 (1.36, 1.48)‡	1.41 (1.35, 1.47)‡
≥1095	2860 (1.7)	1812 (1.1)	1.55 (1.46, 1.65)‡	1.46 (1.37, 1.55)‡	1.43 (1.34, 1.52)‡
Weak anticholinergics					
0	37,441 (22.4)	46,483 (27.8)	1.00	1.00	1.00
1-89	17,588 (10.5)	19,426 (11.6)	1.12 (1.09, 1.15)‡	1.13 (1.10, 1.16)‡	1.13 (1.10, 1.16)‡
90-364	20,781 (12.4)	19,376 (11.6)	1.31 (1.28, 1.34)‡	1.34 (1.31, 1.38)‡	1.34 (1.31, 1.37)‡
365-1094	32,984 (19.7)	31,092 (18.6)	1.29 (1.27, 1.32)‡	1.34 (1.31, 1.37)‡	1.32 (1.29, 1.35)‡
≥1095	58,317 (34.9)	50,734 (30.4)	1.40 (1.37, 1.43)‡	1.43 (1.40, 1.47)‡	1.39 (1.36, 1.43)‡

Abbreviations: CI, confidence interval; DDD, defined daily dose.

* Model 1 was unadjusted; Model 2 was adjusted for sociodemographic factors, healthcare utilization in the previous year, and history of physical and neuropsychiatric comorbidities; Model 3 was additionally adjusted for the use of other medications.

† $p < 0.01$

‡ $p < 0.001$

Table S12. Association between cumulative use of anticholinergic drugs and all-cause dementia, using alternative covariates adjustments

Exposure, DDDs	No. of cases (%)	No. of controls (%)	Odds ratio (95% CI)*		
			Adjust for multimorbidity and polypharmacy	Adjust for drug use during the exposure period	Adjust for covariates at the index date
Strong anticholinergics					
0	157,103 (78.7)	164,771 (82.6)	1.00	1.00	1.00
1-89	24,856 (12.5)	23,161 (11.6)	1.10 (1.08, 1.12)‡	1.08 (1.06, 1.11)‡	1.03 (1.01, 1.06)†
90-364	10,673 (5.3)	7538 (3.8)	1.39 (1.35, 1.43)‡	1.33 (1.28, 1.37)‡	1.27 (1.23, 1.31)‡
365-1094	4763 (2.4)	2880 (1.4)	1.57 (1.50, 1.65)‡	1.44 (1.37, 1.52)‡	1.38 (1.31, 1.45)‡
≥1095	2131 (1.1)	1176 (0.6)	1.66 (1.55, 1.79)‡	1.49 (1.38, 1.60)‡	1.50 (1.39, 1.62)‡
Weak anticholinergics					
0	59,225 (29.7)	63,384 (31.8)	1.00	1.00	1.00
1-89	21,236 (10.6)	19,708 (9.9)	1.10 (1.07, 1.12)‡	1.09 (1.07, 1.12)‡	1.09 (1.07, 1.12)‡
90-364	23,546 (11.8)	21,563 (10.8)	1.10 (1.08, 1.13)‡	1.09 (1.06, 1.11)‡	1.10 (1.07, 1.13)‡
365-1094	34,309 (17.2)	32,962 (16.5)	1.05 (1.03, 1.08)‡	1.03 (1.00, 1.05)	1.06 (1.04, 1.09)‡
≥1095	61,210 (30.7)	61,909 (31.0)	1.00 (0.98, 1.02)	0.94 (0.92, 0.96)‡	1.00 (0.98, 1.02)

Abbreviations: CI, confidence interval; DDD, defined daily dose.

* Compared with Model 3 in the primary analysis, alternative covariates adjustments included: (1) additionally adjusting for multimorbidity evaluated by the Charlson comorbidity index and polypharmacy measured by the count of medications at baseline, (2) additionally adjusting for cumulative use of medications considered as baseline covariates during the exposure period, and (3) adjusting for all covariates ascertained at the index date rather than at baseline.

† $p < 0.01$

‡ $p < 0.001$

Table S13. Association between cumulative use of anticholinergic drugs and all-cause dementia, by incorporating prevalent users

Exposure, DDDs	No. of cases (%)	No. of controls (%)	Odds ratio (95% CI)*		
			Model 1	Model 2	Model 3
Strong anticholinergics					
0	165,029 (71.6)	178,329 (77.4)	1.00	1.00	1.00
1-89	29,448 (12.8)	27,861 (12.1)	1.14 (1.12, 1.16)‡	1.11 (1.09, 1.13)‡	1.10 (1.08, 1.12)‡
90-364	16,587 (7.2)	11,992 (5.2)	1.48 (1.45, 1.52)‡	1.40 (1.37, 1.44)‡	1.37 (1.33, 1.40)‡
365-1094	11,189 (4.9)	7297 (3.2)	1.65 (1.60, 1.70)‡	1.49 (1.45, 1.54)‡	1.45 (1.41, 1.50)‡
≥1095	8196 (3.6)	4970 (2.2)	1.79 (1.72, 1.85)‡	1.54 (1.49, 1.60)‡	1.50 (1.44, 1.56)‡
Weak anticholinergics					
0	65,035 (28.2)	70,671 (30.7)	1.00	1.00	1.00
1-89	24,488 (10.6)	22,846 (9.9)	1.13 (1.10, 1.15)‡	1.12 (1.09, 1.14)‡	1.10 (1.08, 1.13)‡
90-364	27,670 (12.0)	25,448 (11.0)	1.13 (1.11, 1.15)‡	1.12 (1.10, 1.14)‡	1.11 (1.08, 1.13)‡
365-1094	40,334 (17.5)	38,963 (16.9)	1.08 (1.06, 1.10)‡	1.07 (1.05, 1.09)‡	1.06 (1.04, 1.08)‡
≥1095	72,922 (31.6)	72,521 (31.5)	1.04 (1.02, 1.06)‡	1.01 (1.00, 1.03)	1.01 (0.99, 1.03)

Abbreviations: CI, confidence interval; DDD, defined daily dose.

* Model 1 was unadjusted; Model 2 was adjusted for sociodemographic factors, healthcare utilization in the previous year, and history of physical and neuropsychiatric comorbidities; Model 3 was additionally adjusted for the use of other medications.

† $p < 0.01$

‡ $p < 0.001$

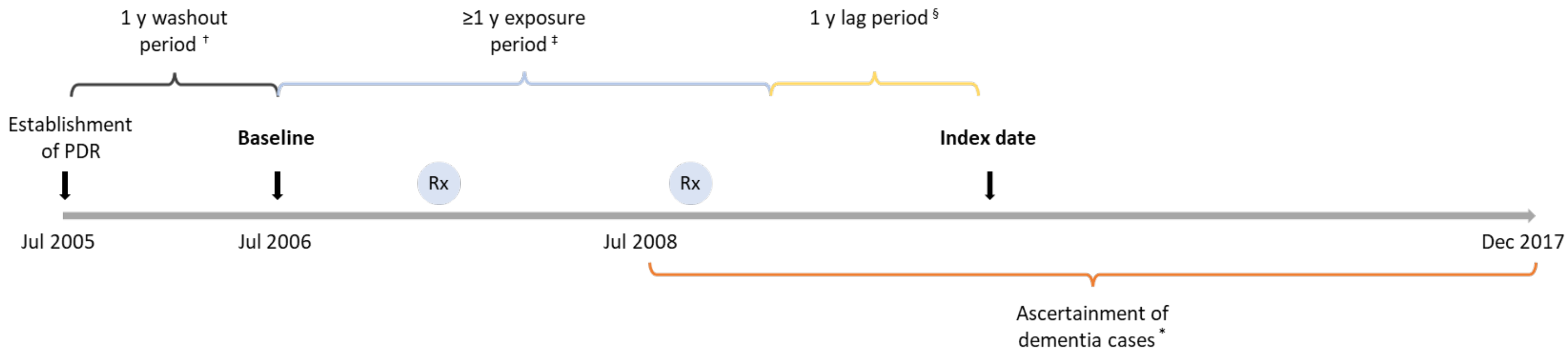

Figure S1. Graphical illustration of the study design

Abbreviations: ACB, Anticholinergic Cognitive Burden; PDR, Swedish Prescribed Drug Register.

* Inclusion criteria: individuals aged ≥40 years with incident dementia between July 1, 2008, and December 31, 2017.

† Excluding individuals who had used drugs with strong anticholinergic effects (an ACB score of 2 or 3) during the 1-year washout period.

‡ A ≥1-year exposure period was ensured to assess the anticholinergic burden.

§ A 1-year lag period was applied to reduce protopathic bias.

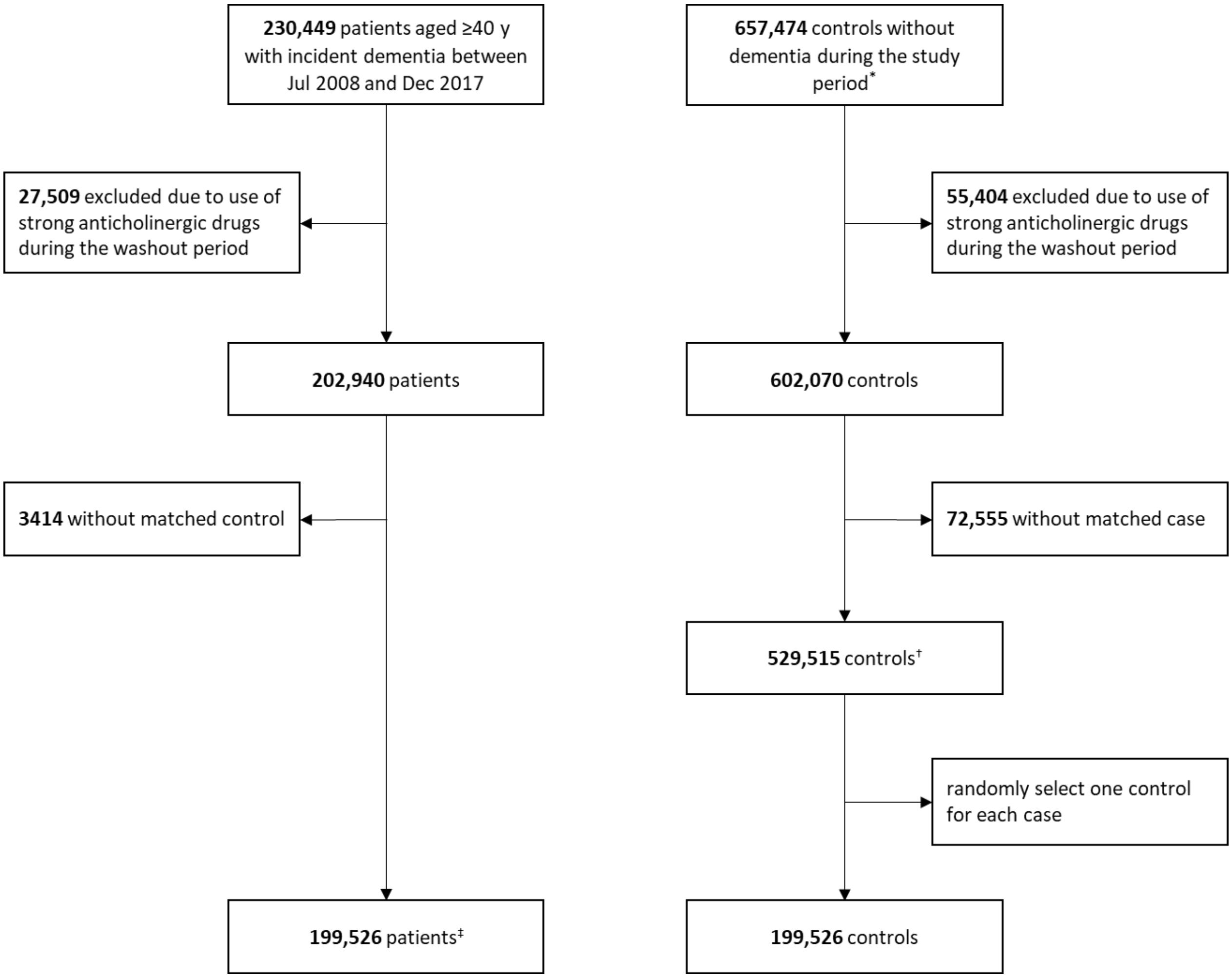

Figure S2. Flowchart for the selection of dementia cases and matched controls

* For each patient with dementia, up to 4 controls without dementia were randomly selected from the Total Population Register, based on year of birth (±3 years), sex, and region of residence.

† A sensitivity analysis was conducted using the entire control sample.

‡ Subtype of dementia was analyzed among 76,613 patients with a specific type of dementia; severity of dementia was analyzed among 58,800 patients with an available Mini-Mental State Examination score.

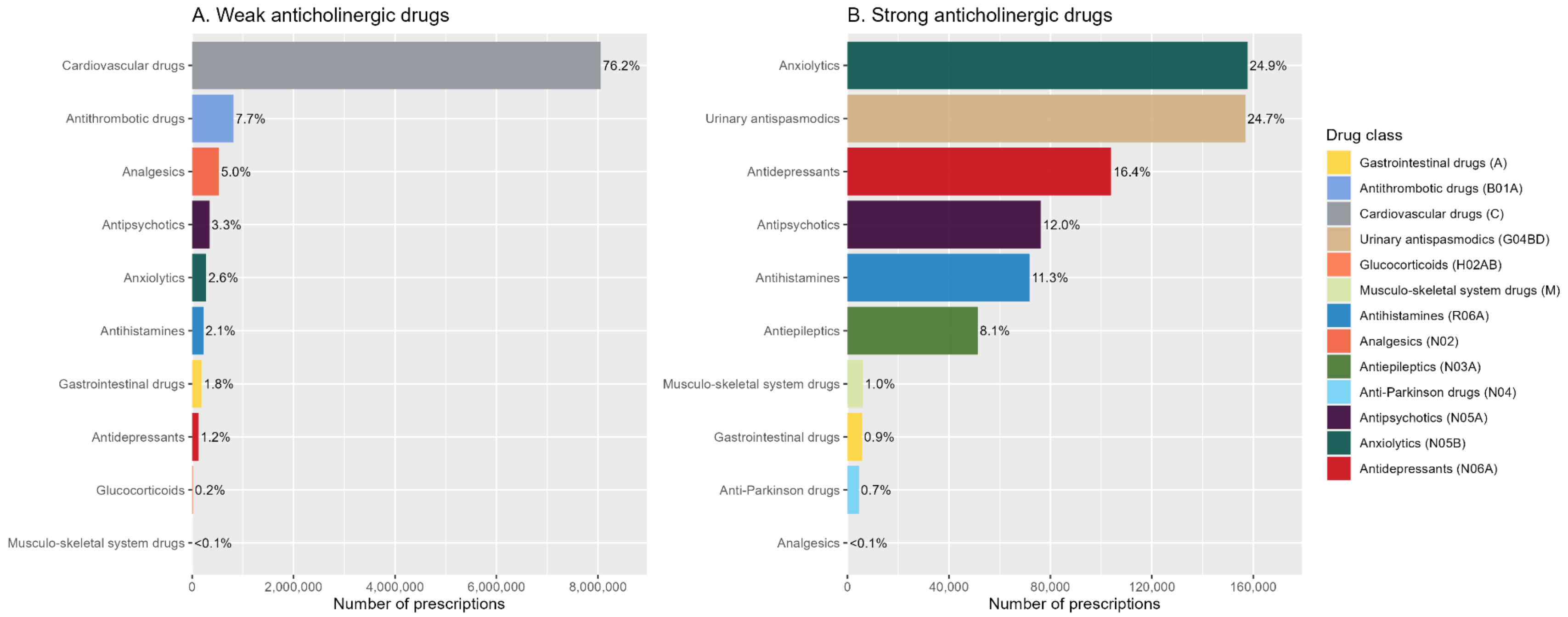

Figure S3. Number of prescriptions during the drug exposure period, by drug class

The scale for weak anticholinergic drugs is 50 times greater than the scale for strong anticholinergic drugs.

The percentage indicates the proportion of the total number of prescriptions by drug class within each level of anticholinergic potency.

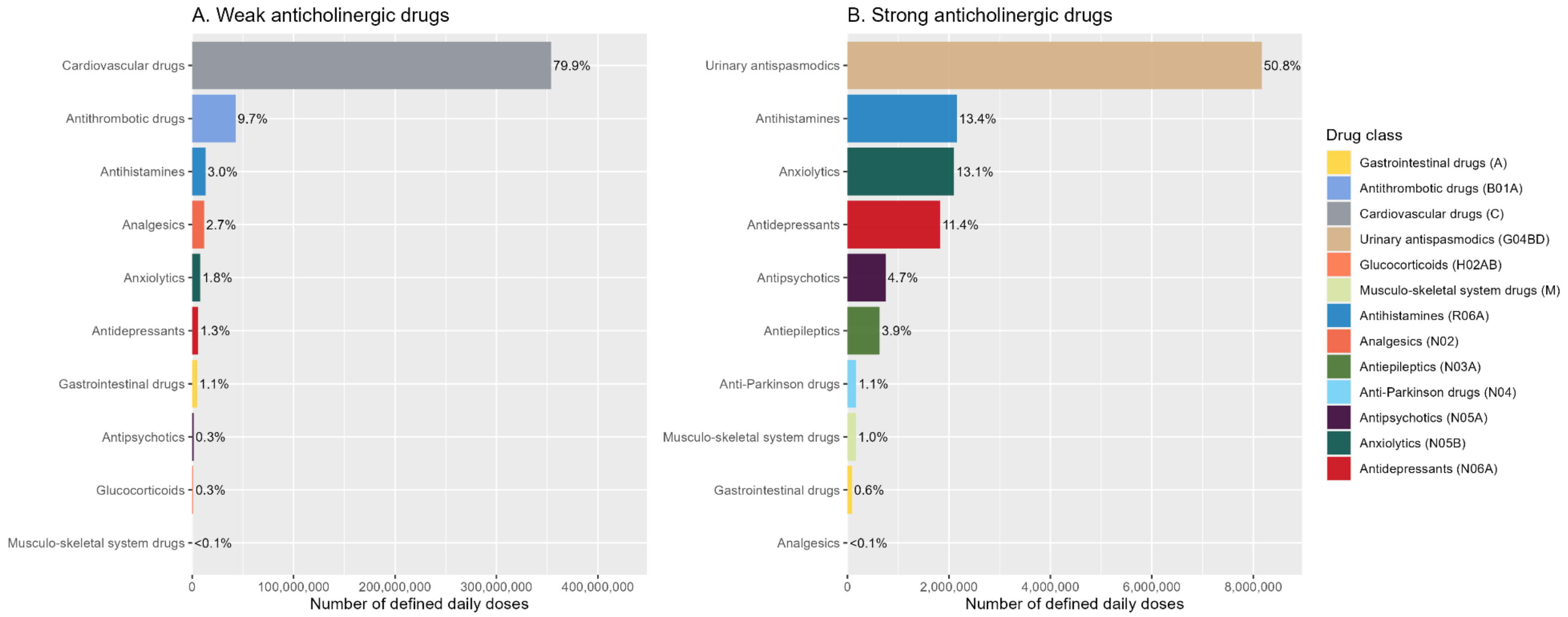

Figure S4. Number of defined daily doses during the drug exposure period, by drug class

The scale for weak anticholinergic drugs is 50 times greater than the scale for strong anticholinergic drugs.

The percentage indicates the proportion of the total number of defined daily doses by drug class within each level of anticholinergic potency.

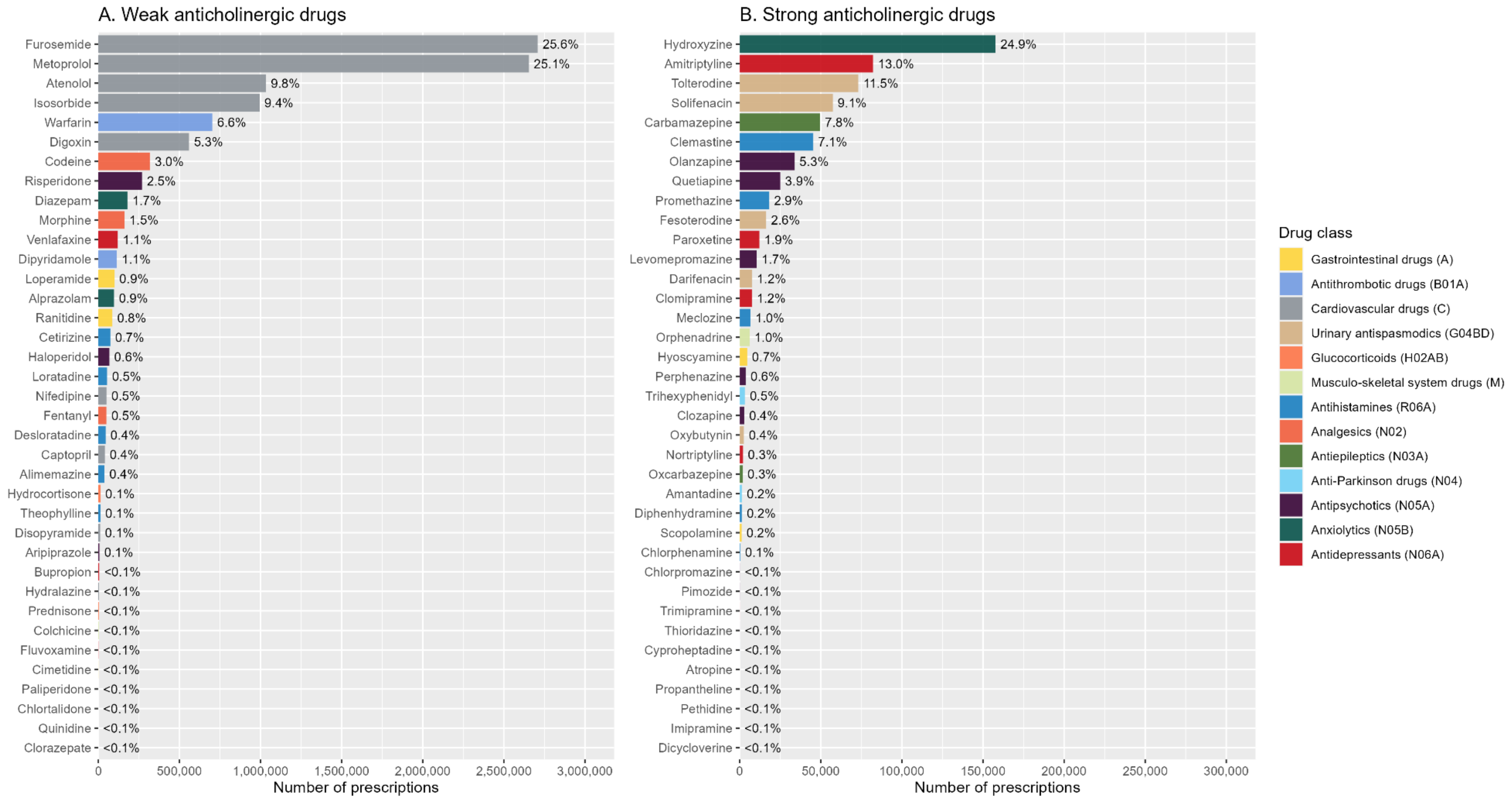

Figure S5. Number of prescriptions during the drug exposure period, by individual drug

The scale for weak anticholinergic drugs is 10 times greater than the scale for strong anticholinergic drugs.
The percentage indicates the proportion of the total number of defined daily doses by individual drug within each level of anticholinergic potency.

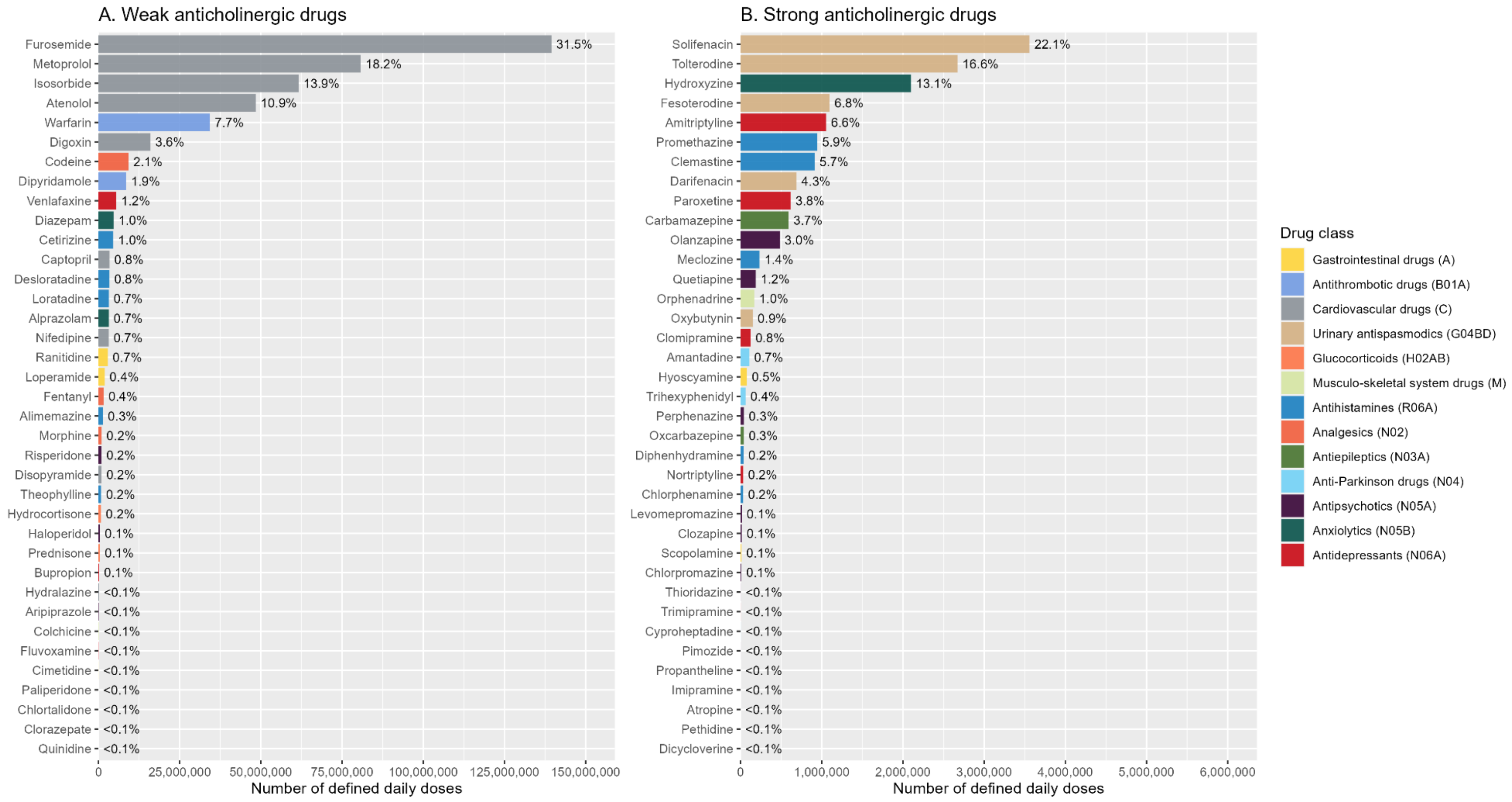

Figure S6. Number of defined daily doses during the drug exposure period, by individual drug

The scale for weak anticholinergic drugs is 25 times greater than the scale for strong anticholinergic drugs.

The percentage indicates the proportion of the total number of defined daily doses by individual drug within each level of anticholinergic potency.

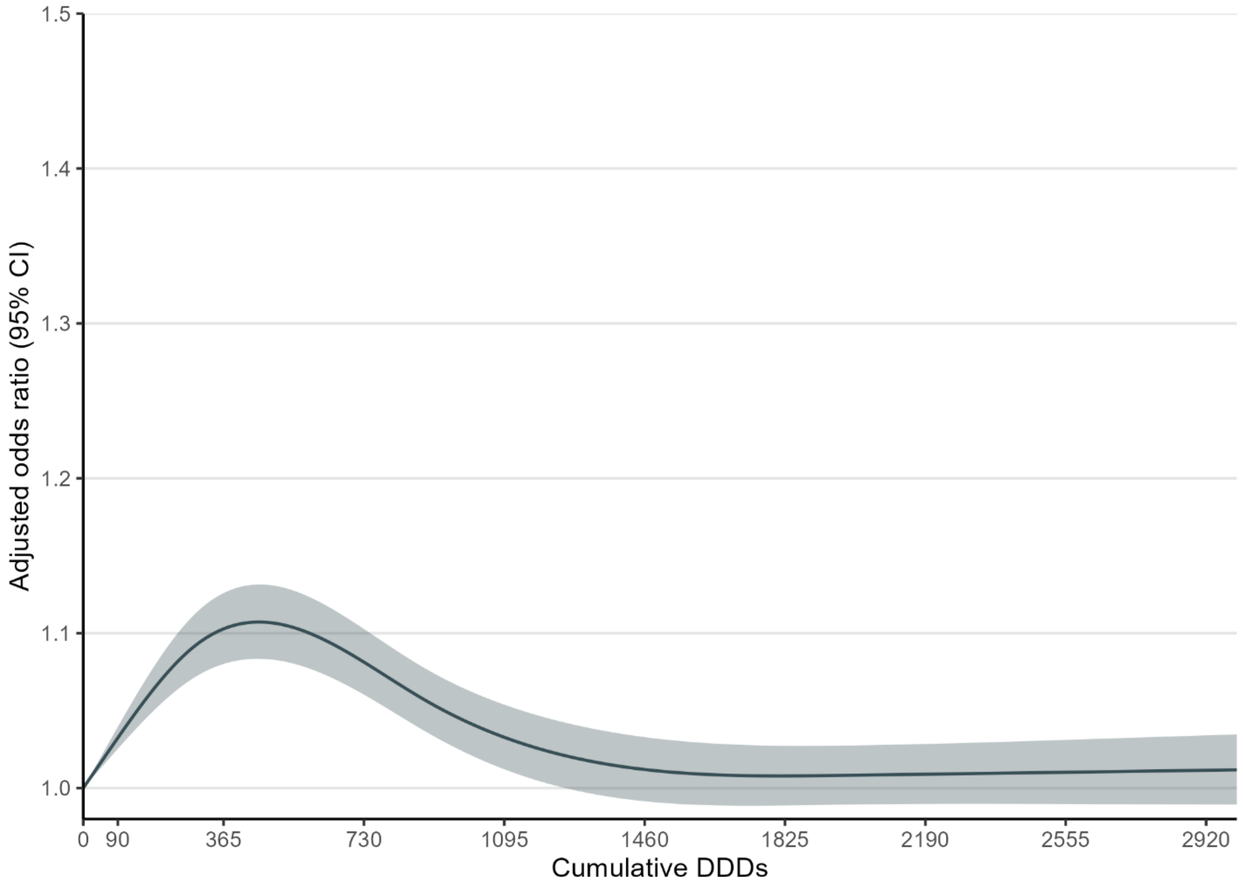

Figure S7. Association between cumulative use of weak anticholinergic drugs and all-cause dementia using restricted cubic splines

Abbreviations: CI, confidence interval; DDD, defined daily dose.

The cubic spline was constructed with knots placed at the 5th, 35th, 65th, and 95th percentiles of cumulative doses. The solid line represents the adjusted odds ratios, and the shaded area represents the corresponding 95% CIs.
